# Refining value-at-risk estimates using a Bayesian Markov-switching GJR-GARCH copula-EVT model

**DOI:** 10.1371/journal.pone.0198753

**Published:** 2018-06-22

**Authors:** Marius Galabe Sampid, Haslifah M. Hasim, Hongsheng Dai

**Affiliations:** Department of Mathematical Sciences, University of Essex, Colchester, United Kingdom; Feng Chia University, TAIWAN

## Abstract

In this paper, we propose a model for forecasting Value-at-Risk (VaR) using a Bayesian Markov-switching GJR-GARCH(1,1) model with skewed Student’s-*t* innovation, copula functions and extreme value theory. A Bayesian Markov-switching GJR-GARCH(1,1) model that identifies non-constant volatility over time and allows the GARCH parameters to vary over time following a Markov process, is combined with copula functions and EVT to formulate the Bayesian Markov-switching GJR-GARCH(1,1) copula-EVT VaR model, which is then used to forecast the level of risk on financial asset returns. We further propose a new method for threshold selection in EVT analysis, which we term the *hybrid* method. Empirical and back-testing results show that the proposed VaR models capture VaR reasonably well in periods of calm and in periods of crisis.

## Introduction

In recent decades, Value-at-Risk (VaR) has become a key tool for measuring market risk; it provides risk managers with a quantitative measure of the downside risk of a firm or investment portfolio during a given time frame. VaR attempts to summarise the total risk in a portfolio of asset or exposures to risk factors in a single number over a target horizon.

There are several methods to estimate VaR; the most commonly used by financial institutions are the variance-covariance, historical simulation and Monte Carlo simulation methods (see [1–3] and the references therein). Historical simulation relies on actual data and is based on the assumption that history will repeat itself; the VaR is estimated by running hypothetical portfolios from historical data [4]. The variance-covariance and Monte Carlo simulation methods assume that asset returns are independent and identically distributed, a major weakness in these VaR models.

Traditional VaR models assume asset returns in financial markets to be normally distributed; thus, changes in asset prices are independent of each other and exhibit constant volatility over time. This is not the case in real life i.e., financial asset returns are leptokurtic and heavy tailed with non-constant volatility [5, 6]. The normality assumption leads to inaccurate estimates in the tails of the distribution and hence of the probability of extreme events, which leads to underestimation of the likelihood of extreme tail losses. This is because the normal distribution has light tails, and VaR attempts to capture the behaviour of the portfolio return in the left tail. A model based on the normal distribution underestimates the frequency of outliers and hence the true VaR [2]. Additionally, the normality assumption implies volatility is constant over time, and recent price changes, which are based on current market information, will be assigned weights in equal proportion to older ones. If the dependence characteristics of the extreme realisations differ from all others in the sample, the consequences might be dire [7].

Non-normality for univariate models is associated with the dependence (i.e., correlation) structure between the asset returns. For multivariate models, non-normality is associated with the joint probability of the univariate models’ marginal probabilities, i.e., the joint probability of large market movements, known as *tail dependence*. Because of the complexity of multivariate distributions, the VaR estimation of a portfolio of assets can be quite difficult. To avoid the normality assumption, extreme value theory (EVT) is often used to model the tail behaviour of asset returns. However, EVT also assumes extreme events to be independent and identically distributed, which might not hold in periods of severe crisis [8]. [9] suggests applying EVT to the noise variables of the return series, which are normally distributed, to obtain the *q*^*th*^ quantile used to estimate conditional, robust VaR estimates. By doing so, the problem of volatility clustering and other related effects, such as excess kurtosis, is accounted for. This approach was further investigated by [4]; they combined a GARCH(1,1) model as the underlying volatility model with EVT to estimate the VaR of the Tunisian stock market index and showed that the GARCH-EVT-based VaR approach appears to be more effective and realistic than traditional VaR estimation methods.

A study by [10] have shown that volatility predictions following econometric models that ignore regime changes and time varying parameters have several drawbacks. For example, they may fail to capture the dynamics of fluctuations in the time series data. Ignoring regime changes and time varying parameters in high-volatility periods causes significant upwards bias in estimating the GARCH parameters, which impairs volatility forecasts [11]. The Markov-switching GARCH model, first developed by [12] and later improved by [11, 13], helps address the issues since it allows the parameters of GARCH models to vary over time according to a latent discrete Markov process, which leads to volatility forecasts that can rapidly adapt to variations [14].

The problem of dependence can also be improved with the help of copula theory, which enables the construction of flexible multivariate distributions with different margins and dependence structures. This allows the joint distribution of the portfolio to be free from assumptions of normality and linear correlation. [15, 16] have demonstrated that VaR estimates obtained by combining GARCH models, EVT and copula functions are more accurate than those obtained using traditional VaR estimation methods or methods that combined copulas with conventionally employed empirical distributions.

In this paper, we combine the Bayesian Markov-switching GJR-GARCH(1,1) model with skewed Student’s-*t* distribution, copula functions and the peaks over threshold (POT) method of EVT to estimate VaR in selected banks in the United Kingdom (UK) using actively traded stocks on the London Stock Exchange.

## Methodology

### Markov-switching GJR-GARCH model

Let *r*_*t*_ represent a time series, then a general Markov-switching GARCH specification can be represented as

$$\begin{array}{c} r_{t}|\left(\Delta_{t}=k,\Omega_{t-1}\right)∼D\left(0,h_{k,t},\Theta_{k}\right), \end{array}$$ (1)

$$\begin{array}{c} r_{t}=\epsilon_{t}\left(h_{\Delta_{t},t}^{\frac{1}{2}}\right), \end{array}$$ (2)

where Δ_*t*_ is a Markov chain (a stochastic variable) defined on the parameter space *S* = {1, …, *K*} that symbolises the model, *D*(0, *h*_*k*, *t*_, Θ_*k*_) is a continuous distribution with zero mean and conditional variance *h*_*k*,*t*_, *ϵ*_*t*_ is the distribution of the noise variables, which assumes a skewed Student’s-*t* distribution, Ω_*t*−1_ is the information set observed up to time *t* − 1, and Θ_*k*_ is a vector of the shape parameters.

We define a *K* × *K* transition probability matrix **P**, with distinctive elements

$$\begin{array}{c} p_{ij}=P\left[\Delta_{t}=j|\Delta_{t-1}=i\right],\;\forall i,j\in \left\{1,\ldots ,K\right\},\;0<p_{ij}<1,\;\Sigma_{j=1}^{K}p_{ij}=1, \end{array}$$ (3)

where *p*_*ij*_ is the probability of transition from state Δ_*t*−1_ = *i* to state Δ_*t*_ = *j*. *k* represents each regime in the Markov chain. The conditional variance, *h*_*k*,*t*_, for *k* = 1, …*K* are assumed to follow *K-separate* GARCH-type processes which evolve in parallel [11, 14]. The Markov switching GARCH models use a stochastic process to define the unknown states [17].

The reliability of a good VaR model depends on the type of volatility model which it incorporates. As discussed above, most financial asset returns are not independently and identically distributed; they exhibit fat tails, leverage effects, and volatility is not constant over time. Volatility reacts differently with large negative returns as compared to positive returns reflecting leverage effects [2]. GARCH models often fail to capture these movements. A good volatility estimator must be able to capture the true behaviour of risk factor returns, it should be easy to implement for a wide range of risk factors, and finally, it should be possible to extent the approach to portfolios with a number of different risk factors [3]. It is well known that traditional GARCH models cannot capture the asymmetric response of volatility. Several other extensions of GARCH models have since been developed as possible solutions to these drawbacks. The most common of these are the exponential generalised ARCH (EGARCH) model [18], the threshold GARCH (TGARCH) model [19], and the GJR-GARCH model [20]. The only significant, albeit minor, difference between TGARCH and GJR-GARCH models is that TGARCH uses standard deviation instead of variance in its specifications [21]. We employ the Markov-switching GARCH model of [11] to capture the differences in the variance dynamics of high and low volatility periods [14], and use the GJR-GARCH(1,1) model to capture the asymmetry response in the conditional volatility process, hence the Markov-switching GJR-GARCH(1,1) model (MS-GJR-GARCH(1,1)).

The conditional variance of a MS-GJR-GARCH model is defined as

$$\begin{array}{c} h_{k,t}=\alpha_{0,k}+\left(\alpha_{1,k}+\alpha_{2,k}I_{\left\{r_{t-1}<0\right\}}\right)r_{t-1}^{2}+\beta_{k}h_{k,t-1},\;k=1,\ldots K, \end{array}$$ (4)

where $I_{\left\{\cdot \right\}}$ is an indicator function introduced to capture the leverage effect such that

$$\begin{array}{c} I_{t-1}=\left\{ \begin{array}{c} 1,\;\text{if}\;r_{t-1}<0,\\ 0,\;\text{if}\;r_{t-1}\geq 0. \end{array}\right. \end{array}$$ (5)

*α*_2,*k*_ controls the degree of asymmetry in the conditional volatility to the past shock in regime *k* [14]. Thus, *α*_2,*k*_ > 0 indicates the presence of leverage effect which implies previous negative returns have higher influence on the volatility. The constraints *α*_0,*k*_ > 0, *α*_1,*k*_ + *α*_2,*k*_ ≥ 0 and *β*_*k*_ ≥ 0 ensures a positive variance while covariance stationary is achieved by ensuring that

$$\begin{array}{c} \alpha_{1,k}+\alpha_{2,k}\text{E}\left[\epsilon_{k,t}^{2}I_{\left\{\epsilon_{k,t}<0\right\}}\right]+\beta_{k}<1, \end{array}$$ (6)

where $I_{\left\{.\right\}}=1$ if the condition holds and 0 otherwise. Note that $\text{E}\left[\epsilon_{k,t}^{2}I_{\left\{\epsilon_{k,t}<0\right\}}\right]=\frac{1}{2}$ when *ϵ*_*k*_ is symmetrically distributed.

For the conditional distribution of *r*_*t*_ in each regime of the Markov chain, we employ a skew and fat tail error probability distribution; the skewed Student’s-*t* distribution. We use the skewed Student’s-*t* distribution because it is able to account for the excess kurtosis in the conditional distribution that is common with financial time series processes [22]. Moreover, recent studies by [23, 24] have shown that skewed Student’s-*t* errors distribution is a good choice, when compared to a range of existing alternatives. The probability density function (PDF) of a Student’s-*t* distribution is defined as

$$\begin{array}{c} f_{s}\left(\epsilon ,\nu \right)=\frac{\Gamma (\frac{\nu +1}{2})}{\sqrt{\left(\nu -2\right)\pi \Gamma \left(\frac{\nu }{2}\right)}}{\left(1+\frac{\epsilon^{2}}{\nu -2}\right)}^{-\frac{\nu +1}{2}},\;\epsilon \in R, \end{array}$$ (7)

where the constraint on the degrees of freedom parameter *ν* > 2 is imposed to guarantee that the second order moment exist, and Γ(⋅) is the Gamma function. Skewness is introduced by an additional parameter *γ*_*k*_ > 0 as defined in [25]; that is

$$\begin{array}{c} p\left(\epsilon_{k}|v,\gamma_{k}\right)=\frac{2}{\gamma_{k}+\frac{1}{\gamma_{k}}}\left\{f_{s}\left(\frac{\epsilon_{k}}{\gamma_{k}}\right)I_{\left[0,\infty \right)}\left(\epsilon_{k}\right)+f_{s}\left(\gamma_{k}\epsilon_{k}\right)I_{\left(-\infty ,0\right)}\left(\epsilon_{k}\right)\right\}. \end{array}$$ (8)

When *γ*_*k*_ ≠ 1, the posterior distribution, *p*(*ϵ*_*k*_|*v*, *γ*_*k*_) loses symmetry (see [14, 25, 26] for more details on skewed Student’s-*t* probability distribution).

We use Bayesian statistics to estimate the posterior distribution of the variance equation because the Bayesian estimation method provides reliable results even for finite samples. Moreover, it is usually straightforward when using the Bayesian estimation method, to obtain the posterior distribution of any non-linear function of the model parameter. By comparison, when using the classical maximum likelihood method, it is not easy to perform inferences on non-linear functions of the model parameters, while the convergence rate is slow and presents limitations when the residuals are heavy tailed. The constraints on the GARCH parameters to guarantee a positive variance can be incorporated via priors whereas the classical maximum likelihood method may impede some optimisation procedures [27, 28].

We define a vector of the risk factor returns as **r** = (*r*_1_, …, *r*_*T*_)′, *θ*_*k*_ = (*α*_0,*k*_, *α*_1,*k*_, *α*_2,*k*_, *β*_*k*_, P)′, and a vector of the model parameters as Λ = (*θ*_1_, Θ_1_, …, *θ*_*K*_, Θ_*K*_); Θ_*K*_ = (*ν*_*K*_, *γ*_*K*_). Then, from Bayes theorem and prior distribution of the model parameters *p*(Λ), we have

$$\begin{array}{c} p_{ij}=\mathrm{Pr}\left[\Delta_{t}=j|\Delta_{t-1}=i\right]=\frac{f\left(r_{t}|\Delta_{t}=j,\Omega_{t-1};\Lambda \right)\mathrm{Pr}\left(\Delta_{t}=j|\Omega_{t-1}\right)}{\Sigma_{i=1}^{k}f\left(r_{t}|\Delta_{t}=i,\Omega_{t-1};\Lambda \right)\mathrm{Pr}\left(\Delta =i|\Omega_{t-1}\right)}, \end{array}$$ (9)

where *f*(*r*_*t*_|Δ_*t*_ = *j*, Ω_*t*−1_; Λ) is the conditional probability density of *r*_*t*_ at time *t* restrictive on Ω_*t*−1_ and regime *j*. Therefore we have

$$\begin{array}{c} f\left(r_{t}|\Lambda ,\Omega_{t-1}\right)=\sum_{i=1}^{k}\sum_{j=1}^{k}\mathrm{Pr}\left[\Delta_{t}=j|\Delta_{t-1}=i\right]f_{D}\left(r_{t}|\Delta_{t}=j,\Omega_{t-1};\Lambda \right) \end{array}$$ (10)

and a likelihood function

$$\begin{array}{c} L\left(\Lambda |r\right)=\prod_{t=1}^{T}f\left(r_{t}|\Lambda ,\Omega_{t-1}\right). \end{array}$$ (11)

The Metropolis Hasting (MH) algorithm of Markov Chain Monte Carlo (MCMC) is then employed to estimate the parameter values of the posterior distribution. As discussed in [22], because of the recursive nature of the variance equation, the prior density *p*(Λ) and posterior density *p*(**r**|Λ) do not belong to the same distributional family and, consequently, cannot be expressed in close form. The MH algorithm allows draws to be generated from any density and whose normalising constant is unknown.

In the MH algorithm, Λ is a random variable with Markov chains generated as (Λ^[0]^), …, (Λ^[*j*]^), … in a parameter space. As the number of realised chains reaches infinity, *p*(**r**|Λ) tends to a normalised probability distribution with a random variable (Λ^[*j*]^) [29]. The chain converges to its stationary distribution and the optimal mean values of the posterior distribution parameters are realised. [22] summarises the MH algorithm as follows: (i) Initialise the iteration counter to *j* = 1 and set the initial value Λ^[0]^. (ii) Move the chain to a new value Λ^⋆^ generated from a proposal density *q*(⋅|Λ^[*j*−1]^). (iii) Evaluate the acceptance probability of the move from Λ^[*j*−1]^ to Λ^[⋆]^ given by

$$\begin{array}{c} \min\{\frac{p(\Lambda^{⋆}|r)}{p(\Lambda^{\left[j-1\right]}|r)}\frac{q(\Lambda^{\left[j-1\right]}|\Lambda^{⋆})}{q(\Lambda^{⋆}|\Lambda^{\left[j-1\right]})},1\}. \end{array}$$

If the move is accepted, set Λ^[*j*]^ = Λ^⋆^; if not, set Λ^[*j*]^ = Λ^[*j*−1]^ (i.e., the chain does not move). If chosen from a symmetric proposal density, i.e., *q*(Λ^[*j*]^|Λ^⋆^) = *q*(Λ^⋆^|Λ^[*j*]^), then the acceptance probability reduces to

$$\begin{array}{c} \min\{\frac{p(\Lambda^{⋆}|r)}{p(\Lambda^{\left[j\right]}|r)},1\}. \end{array}$$

(iv) Finally, change the counter from *j* to *j* + 1 and go back to step (ii) until convergence is reached. More details on MH algorithms can be found in [30–33].

### Copula theory

Copula theory enables the construction of a flexible multivariate distribution with varying margins and dependence structures; it is free from assumptions of normality or linear correlation. In addition, copulas can easily capture the tail dependence of asset returns, i.e., the joint probability of large market movements.

Copula theory was first developed by [34] to describe the dependence structure between random variables. It was later introduced to the finance literature by [35, 36]. Consequently, [37] introduced the application of copula theory to financial asset returns, and [38] expanded the framework of copula theory with respect to the time-varying nature of financial dependence schemes. Copula theory has also been used in risk management to measure the VaR of portfolios, including both unconditional [39–41] and conditional distributions [42–44].

In multivariate settings, we use the following version of Sklar’s theorem as given by [41] for the purpose of VaR estimation:

Sklar’s theorem: Consider an *n*-dimensional joint distributional function *F*(*x*), with uniform margins *F*_1_(*x*_1_), …, *F*_*n*_(*x*_*n*_); *x* = (*x*_1_, …, *x*_*n*_), with −∞ ≤ *x*_*i*_ ≤ ∞, then there exists a copula *C*: [0, 1]^*n*^ → [0, 1] such that

$$\begin{array}{c} F\left(x_{1},\ldots ,x_{n}\right)=C\left(F_{1}\left(x_{1}\right),\cdots ,F_{n}\left(x_{n}\right)\right), \end{array}$$ (12)

determined under absolute continuous margins as

$$\begin{array}{c} C\left(u_{1},\ldots ,u_{n}\right)=F\left(F_{1}^{-1}\left(u_{1}\right),\cdots ,F_{n}^{-1}\left(u_{n}\right)\right), \end{array}$$ (13)

otherwise, *C* is uniquely determined on the range *R*(*F*_1_) × … × *R*(*F*_*n*_). Equally, if *C* is a copula and *F*_1_, …, *F*_*n*_ are univariate distribution functions, then Eq (12) is a joint distribution function with margins *F*_1_, …, *F*_*n*_ [45].

The copula *C*(*u*_1_, …, *u*_*n*_) has density *c*(*u*_1_, …, *u*_*n*_) associated to it and defined as

$$\begin{array}{c} c\left(u_{1},\ldots ,u_{n}\right)=\frac{\partial_{n}C\left(u_{1},\ldots ,u_{n}\right)}{\partial u_{1},\ldots ,\partial u_{n}} \end{array}$$ (14)

and is related to the density function *F* for continuous random variables denoted as *f*, by the canonical copula representation [16]

$$\begin{array}{c} f\left(x_{1},\ldots ,x_{n}\right)=c\left(F_{1}\left(x_{1}\right),\ldots ,F_{n}\left(x_{n}\right)\right)\prod_{i=1}^{n}f_{i}\left(x_{i}\right), \end{array}$$ (15)

where *f*_*i*_ are the marginal densities that can be different from each other [41, 43, 45, 46].

[16, 47] discuss two commonly used families of copulas in financial applications: the elliptical and the Archimedean copulas.

Elliptical copulas are derived from the elliptical distribution by applying Sklar’s theorem. The most common are the Gaussian and the Student’s-*t* copulas, which are symmetric. Their dependence structure is determined by a standardised correlation or dispersion matrix because of the invariant property of copulas. Consider a symmetric positive definite matrix *ρ* with *diag*(*ρ*) = (1, 1, …, 1)^*T*^; we can represent the multivariate Gaussian copula (MGC) as

$$\begin{array}{c} C_{\rho }^{G_{a}}=\Phi_{\rho }\left(\Phi^{-1}\left(u_{1}\right),\ldots ,\Phi^{-1}\left(u_{n}\right)\right), \end{array}$$ (16)

where Φ_*ρ*_ is the standardised multivariate normal distribution and $\Phi_{\rho }^{-1}$ is the inverse standard univariate normal distribution function of *u* with correlation matrix *ρ*. If the margins are normal, then the Gaussian copula will generate the standard Gaussian joint distribution function with density function

$$\begin{array}{cc} c_{\rho }^{G_{a}}\left(u_{1},u_{2},\ldots ,u_{n}\right) & =\frac{1}{{\left|\rho \right|}^{\frac{1}{2}}}\exp\left(-\frac{1}{2}\varsigma^{{}^{\prime }}\left(\rho^{-1}-\text{I}\right)\varsigma \right), \end{array}$$ (17)

where $\varsigma ={\left(\Phi^{-1}\left(u_{1}\right),\ldots ,\Phi^{-1}\left(u_{n}\right)\right)}^{{}^{\prime }}$ and I is the identity matrix.

On the other hand, the multivariate Student’s-*t* copula (MTC) has the form

$$\begin{array}{c} T_{\rho ,v}\left(u_{1},\ldots ,u_{n}\right)=t_{\rho ,v}\left(t_{v}^{-1}\left(u_{1}\right),\ldots ,t_{v}^{-1}\left(u_{n}\right)\right) \end{array}$$ (18)

with density function

$$\begin{array}{c} c_{\rho ,v}\left(u_{1},\ldots ,u_{n}\right)={\left|\rho \right|}^{-\frac{1}{2}}\frac{\Gamma (\frac{v+n}{2})}{\Gamma (\frac{v}{2})}{\left(\frac{\Gamma (\frac{v}{2})}{\Gamma (\frac{v+1}{2})}\right)}^{n}\frac{{\left(1+\frac{1}{v}\varsigma^{{}^{\prime }}\rho^{-1}\varsigma \right)}^{-\frac{v+n}{2}}}{\prod_{j=1}^{n}{\left(1+\frac{\varsigma_{j}^{2}}{v}\right)}^{-\frac{v+1}{2}}}, \end{array}$$ (19)

where *t*_*ρ*,*v*_ is the standardised Student’s-*t* distribution with correlation matrix *ρ* and *v* degrees of freedom.

Archimedean copulas are useful in risk management analysis because they capture asymmetric tail dependencies between financial asset returns. The most common are the Gumbel [48], Clayton [49] and Frank [50] copulas [51]. These copulas are built via a generator as

$$\begin{array}{c} C\left(u_{1},\ldots ,u_{n}\right)=\varphi^{-1}\left(\varphi \left(u_{1}\right)+\ldots +\varphi \left(u_{n}\right)\right) \end{array}$$ (20)

with density function

$$\begin{array}{c} c\left(u_{1},\ldots ,u_{n}\right)=\varphi^{-1}\left(\varphi \left(u_{1}\right)+\ldots +\varphi \left(u_{n}\right)\right)\prod_{i=1}^{n}\varphi^{{}^{\prime }}\left(u_{i}\right), \end{array}$$ (21)

where *φ* is the copula generator and *φ*^−1^ is completely monotonic on [0, ∞]. That is, *φ* must be infinitely differentiable with derivatives of ascending order and alternative sign such that *φ*^−1^(0) = 1 and lim_*x* → +∞_ *φ*(*x*) = 0 [47]. Thus, *φ*′(*u*)<0 (i.e., *φ* is strictly decreasing) and *φ*′′(*u*) > 0 (i.e., *φ* is strictly convex).

The Gumbel copula captures upper tail dependence, is limited to positive dependence, and has generator function *φ*(*u*) = (−ln(*u*))^*α*^ and generator inverse $\varphi^{-1}\left(x\right)=\exp\left(-x^{\frac{1}{\alpha }}\right)$. This will generate a Gumbel *n*-copula represented by

$$\begin{array}{c} C\left(u_{1},\ldots ,u_{n}\right)=\exp\left\{-{\left[\sum_{i=1}^{n}{\left(-\ln u_{i}\right)}^{\alpha }\right]}^{\frac{1}{\alpha }}\right\}\;\alpha >1. \end{array}$$ (22)

The generator function for the Clayton copula is given by *φ*(*u*) = *u*^−*α*^ − 1 and generator inverse $\varphi^{-1}\left(x\right)={\left(x+1\right)}^{-\frac{1}{\alpha }}$, which yields a Clayton *n*-copula represented by

$$\begin{array}{c} C\left(u_{1},\ldots ,u_{n}\right)={\left[\sum_{i=1}^{n}u_{i}^{-\alpha }-n+1\right]}^{-\frac{1}{\alpha }}\;\alpha >0. \end{array}$$ (23)

Frank copula has generator function $\varphi \left(u\right)=\ln\left(\frac{\exp(-\alpha u)-1}{\exp(-\alpha )-1}\right)$ and generator inverse $\varphi^{-1}\left(x\right)=-\frac{1}{\alpha }\ln\left(1+e^{x}\left(e^{-\alpha }-1\right)\right)$, which will result in a Frank *n*-copula represented by

$$\begin{array}{c} C\left(u_{1},\ldots ,u_{n}\right)=-\frac{1}{\alpha }\ln\left\{1+\frac{\prod_{i=1}^{n}\left(e^{-\alpha u_{i}}-1\right)}{{\left(e^{-\alpha }-1\right)}^{n-1}}\right\}\;\alpha >0. \end{array}$$ (24)

We follow [52] and employ Gaussian, Student’s-*t*, Gumbel, Frank and Clayton copulas in this study.

### Modelling dependence

The traditional way to measure the relationship between markets and risk factors is to look at their linear correlations, which depend both on the marginal and joint distributions of the risk factors. If there is a non-linear relationship (i.e., in the case of non-normality) the results might be misleading [47]. In this situation, non-parametric invariant measures that are not dependent on marginal probability distributions such as Kendall’s *τ* or Spearman’s *ρ* are more appropriate. Copulas measure a form of dependence between pairs of risk factors (i.e., asset returns) known as concordance using these invariant measures.

Two observations (*x*_*i*_, *y*_*i*_) and (*x*_*j*_, *y*_*j*_) from a vector (*X*, *Y*) of continuous random variables are concordant if (*x*_*i*_ − *x*_*j*_)(*y*_*i*_ − *y*_*j*_) > 0 and discordant if (*x*_*i*_ − *x*_*j*_)(*y*_*i*_ − *y*_*j*_) < 0. Large values of *X* are paired with large values of *Y* and small values of *X* are paired with small values of *Y* as the proportion of concordant pairs in the sample increases. On the other hand, the proportion of concordant pairs decreases as large values of *X* are paired with small values of *Y* and small values of *X* are paired with large values of *Y* [53].

Consider *n* paired continuous observations (*x*_*i*_, *y*_*i*_) ranked from smallest to largest, with the smallest ranked 1, the second smallest ranked 2, and so on. Then, Kendall’s *τ* is defined as the sum of the number of concordant pairs minus the sum of the number of discordant pairs divided by the total number of pairs, i.e., the probability of concordance minus the probability of discordance:

$$\begin{array}{c} \tau_{X,Y}=\mathrm{Pr}\left[\left(x_{i}-x_{j}\right)\left(y_{i}-y_{j}\right)>0\right]-\mathrm{Pr}\left[\left(x_{i}-x_{j}\right)\left(y_{i}-y_{j}\right)<0\right]=\frac{C-D}{C+D}, \end{array}$$ (25)

where *C* is the number of concordant pairs below a particular rank that are larger in value than that particular rank, and *D* is the number of discordant pairs below a particular rank that are smaller in value than that particular rank.

Spearman’s *ρ*, on the other hand, is defined as the probability of concordance minus the probability of discordance of the pair of vectors (*x*_1_, *y*_1_) and (*x*_2_, *y*_3_) with the same margins. That is,

$$\begin{array}{c} \rho_{X,Y}=3\left(\mathrm{Pr}\left[\left(x_{1}-x_{2}\right)\left(y_{1}-y_{3}\right)>0\right]-\mathrm{Pr}\left[\left(x_{1}-x_{2}\right)\left(y_{1}-y_{3}\right)\right]<0\right). \end{array}$$

The joint distribution function of (*x*_1_, *y*_1_) is *H*(*x*, *y*), while the joint distribution function of (*x*_2_, *y*_3_) is *F*(*x*)*G*(*y*) because *x*_2_ and *y*_3_ are independent [54]. Alternatively,

$$\begin{array}{c} \rho_{X,Y}=1-\frac{6\sum_{i=1}^{n}d_{i}^{2}}{n(n^{2}-1)}, \end{array}$$

where *d* is the difference between the ranked samples.

A study by [54] has shown that Kendall’s *τ* and Spearman’s *ρ* depend on the vectors (*x*_1_, *y*_1_), (*x*_2_, *y*_2_) and (*x*_1_, *y*_1_), (*x*_2_, *y*_3_), respectively, through theirs copulas *C*, and that the following relationship holds:

$$\begin{array}{c} \tau_{X,Y}=4\int_{0}^{1}\int_{0}^{1}C\left(u,v\right)dC\left(u,v\right)-1 \end{array}$$

and

$$\begin{array}{c} \rho_{X,Y}=12\int_{0}^{1}\int_{0}^{1}C\left(u,v\right)dudv-3. \end{array}$$

### Extreme value theory

EVT is a statistical approach for estimating extreme events with low frequency but high severity. This technique is widely used in financial risk management since empirical evidence from various studies [5, 6] show that in the majority of cases, financial asset return distributions are heavy-tailed, especially in times of financial instability.

There are two fundamental approaches for modeling extreme events with low frequency but high severity: the block maxima method and the POT method. The POT method is a commonly used method to model extreme events in financial time series data. On the other hand, the block maxima method is not commonly used for statistical inference on financial time series data for a few reasons: (i) The method does not make sufficient use of data as it uses only the sub-period maxima, (ii) the choice of sub-period length is not clearly defined, and (iii) the method is unconditional and does not take into account the effects of other explanatory variables [55]. In this paper we use the POT method based on the generalised Pareto distribution (GPD). The POT method focuses on modeling the exceedances of the losses above a certain threshold *ϑ* and the time of occurrence. The threshold is selected such that there are enough data points to carry out a meaningful statistical analysis. Techniques for selecting the proper threshold are discussed below.

Let ${\left\{x_{i}\right\}}_{i=1}^{T}$ represent the loss variables of an asset return, then as *T* → ∞, ${\left\{x_{i}\right\}}_{i=1}^{T}$ is assumed to be independent and identically distributed, and (*x* − *μ*)/*σ* follows a generalised extreme value (GEV) distribution:

$$\begin{array}{cc} F_{\xi ,\mu ,\sigma }\left(x\right) & =\{\begin{array}{cc} \exp[-{\left(1+\xi x\right)}^{-1/\xi }] & \text{for}\;\xi \neq 0,\\ \exp[-e^{-x}] & \text{for}\;\xi =0, \end{array} \end{array}$$ (26)

where *ξ* is the shape parameter and 1/*ξ* is the tail index of the GEV distribution. *x* < −1/*ξ* if *ξ* < 0 and *x* > −1/*ξ* if *ξ* > 0. Also, let the conditional distribution of the excesses over the threshold, i.e., *x*_*i*_ − *ϑ* = *y*|*x*_*i*_ > *ϑ*, then

$$\begin{array}{c} \mathrm{Pr}\left(x-\vartheta \leq y|x>\vartheta \right)=\frac{\mathrm{Pr}(\vartheta \leq x\leq y+\vartheta )}{\mathrm{Pr}(x>\vartheta )}=\frac{\mathrm{Pr}(x\leq y+\vartheta )-\mathrm{Pr}(x\leq \vartheta )}{1-\mathrm{Pr}(x\leq \vartheta )} \end{array}$$ (27)

$$\begin{array}{c} =\frac{F(y+\vartheta )-F(\vartheta )}{1-F(\vartheta )}=F_{\vartheta }\left(y\right). \end{array}$$ (28)

Again, as *T* → ∞, (*y* + *ϑ* − *μ*)/*σ* follows a GEV distribution; see Eq (26). Therefore,

$$\begin{array}{ccc} \mathrm{Pr}(x-\vartheta \leq y|x>\vartheta ) & = & \frac{F(y+\vartheta )-F(\vartheta )}{1-F(\vartheta )}\\ & = & \frac{\exp\left[-{\left(1+\frac{\xi (y+\vartheta -\mu )}{\sigma }\right)}^{-1/\xi }\right]-\exp\left[-{\left(1+\frac{\xi (\vartheta -\mu )}{\sigma }\right)}^{-1/\xi }\right]}{1-\exp[-{\left(1+\frac{\xi (\vartheta -\mu )}{\sigma }\right)}^{-1/\xi }]}\\ & \approx & 1-{\left(1+\frac{\xi y}{\sigma +\xi (\vartheta -\mu )}\right)}^{-1/\xi }, \end{array}$$ (29)

where *y* > 0 and *σ* + *ξ*(*ϑ* − *μ*) > 0. Let *ψ*(*ϑ*) = *σ* + *ξ*(*ϑ* − *μ*), then as *ϑ* → ∞, Eq (29) is approximated by the GPD

$$\begin{array}{c} G_{\xi ,\psi (\vartheta )}\left(y\right)=\{\begin{array}{cc} 1-{\left[1+\frac{\xi y}{\psi (\vartheta )}\right]}^{-1/\xi } & \text{for}\;\xi \neq 0,\\ 1-\exp[-y/\psi (\vartheta )] & \text{for}\;\xi =0, \end{array} \end{array}$$ (30)

with shape parameter *ξ* and scale parameter *ψ*(*ϑ*), where *ψ*(*ϑ*) > 0, *y* ∈ [0, *x* − *ϑ*] when *ξ* ≥ 0, and $y\in [0,-\frac{\psi (\vartheta )}{\xi }]$ when *ξ* < 0. If *ξ* = 0, then Eq (30) becomes an exponential distribution with parameter 1/*σ* ([55]). Let *y* = *x* − *ϑ*, then Eq (28) can be written as

$$\begin{array}{c} \frac{F(y+\vartheta )-F(\vartheta )}{1-F(\vartheta )}=\frac{F(x)-F(\vartheta )}{1-F(\vartheta )}\approx G_{\xi ,\psi (\vartheta )}\left(x-\vartheta \right) \end{array}$$ (31)

$$\begin{array}{c} ⟹F\left(x\right)=F\left(\vartheta \right)+\left[1-F\left(\vartheta \right)\right]G_{\xi ,\psi (\vartheta )}\left(x-\vartheta \right). \end{array}$$ (32)

The tail estimator for the underlying distribution *F*(*x*|*ξ*, *ψ*(*ϑ*)) is constructed using an empirical estimate of *F*(*ϑ*), i.e., $\hat{F}\left(\vartheta \right)=\left(T-N_{\vartheta }\right)/T$ as

$$\begin{array}{c} \hat{F}\left(x|\xi ,\psi \left(\vartheta \right)\right)\approx \frac{T-N_{\vartheta }}{T}{\left[1+\frac{\hat{\xi }\left(x-\vartheta \right)}{\hat{\psi }\left(\vartheta \right)}\right]}^{-1/\hat{\xi }}, \end{array}$$ (33)

where *N*_*ϑ*_ is the number of observations above the threshold. We obtain the *q*^*th*^ quantile $F_{q}^{-1}={VaR}_{q}$, by inverting Eq (33), for any given small upper tail probability *p* for VaR estimation as

$$\begin{array}{c} {VaR}_{q}=\vartheta -\frac{\hat{\psi }\left(\vartheta \right)}{\hat{\xi }}\left\{1-{\left[\frac{T}{N_{\vartheta }}\left(1-q\right)\right]}^{-\hat{\xi }}\right\}, \end{array}$$ (34)

where *q* = 1 − *p* [4, 55, 56].

After deciding on the choice of *ϑ*, and assuming that the number of points above *ϑ* are independent and identically distributed, the parameters *ψ*(*ϑ*) and *ξ* can be estimated by means of maximum likelihood estimation with likelihood function

$$\begin{array}{c} L\left(x_{i},\ldots ,x_{N_{\vartheta }}|\xi ,\sigma ,\mu \right)=\prod_{i=1}^{N_{\vartheta }}f\left(x_{i}\right)\;\text{for}\;x_{i}>\vartheta . \end{array}$$ (35)

The choice of a threshold *ϑ* is an important step in the POT method because Eq (34) is dependent on *ϑ* and the number of points (i.e., exceedances) above *ϑ* since the parameters are estimated based on the exceedances. Thus, it is very important to find the proper threshold value. There is no clear-cut or wholly satisfactory method to determine a proper threshold that has been determined to date. [57] developed a semi-parametric estimator for the tails of the distribution that estimated the threshold of the bootstrap approximation of the mean square error (MSE) of the tail index and by minimising MSE through the choice of the threshold. [58] further used a two-step subsample bootstrap method to determine the threshold that minimised the asymptotic MSE. [59, 60] propose graphical tools to identify the proper threshold known as the Hill plot and the mean excess plot, respectively. In this paper, we use the mean excess plot and propose its extension, which wee call a *hybrid* method as will be discussed later.

A mean excess function of *x* over a certain threshold *ϑ* is defined as

$$\begin{array}{c} e\left(\vartheta \right)=\text{E}\left(x-\vartheta |x>\vartheta \right)=\frac{\sigma +\xi \vartheta }{1-\xi }. \end{array}$$ (36)

A property of the GPD states that if the excess distribution of *x* given a threshold *ϑ*_0_ be a GPD with shape parameter *ξ* and scale parameter *ψ*(*ϑ*_0_), then for any random threshold *ϑ* > *ϑ*_0_, the excess distribution over the threshold *ϑ* has a GPD with shape parameter *ξ* and scale parameter *ψ*(*ϑ*) = *ψ*(*ϑ*_0_)+*ξ*(*ϑ* − *ϑ*_0_), where 0 < *ξ* < 1 [55]. Then

$$\begin{array}{c} e\left(\vartheta \right)=\text{E}\left(x-\vartheta |x>\vartheta \right)=\frac{\psi \left(\vartheta_{0}\right)+\xi \left(\vartheta -\vartheta_{0}\right)}{1-\xi }, \end{array}$$ (37)

which is a linear function of *ϑ* − *ϑ*_0_ with slope *ξ*/(1 − *ξ*) for *ϑ* > *ϑ*_0_. From the ordered sample {*x*_*i*_}, we calculate and plot the mean excess function, i.e., Eq (37) against each chosen *ϑ*_*i*_ for *ϑ*_*i*_ > *ϑ*_0_. The threshold *ϑ* is then identified as the lowest point on the mean excess plot above which the graph appears to be approximately linear. However, the choice of *ϑ* from the mean excess plot is subjective [8, 55] and might differ from one bank to another using the same data because of different risk tolerances. Different *ϑ* values will give different estimates of the shape and scale parameter. A very high threshold will result in too few data points in the left tail for any meaningful statistical analysis. In contrast, a very low threshold will result in a number of data points above the threshold lying close to the body of the sample data. This will result in a poor approximation because the GPD is a limiting distribution as *ϑ* → ∞; data beyond the threshold will deviate from the GPD since the GPD is not a good approximation for the body of the sample data [8, 56]. We propose a *hybrid* method for selecting a proper threshold value that will significantly diminish the possibility of different *ϑ* values with the same data.

### Data

The data employed in this analysis consist of 2870 daily observations of stock prices actively traded on the London Stock Exchange. The stocks belong to the banking sector and of the top five banks in the UK, i.e., HSBC Holdings, Lloyds Banking Group, Barclays Plc., Royal Bank of Scotland Group, and Standard Chartered Plc. We refer to these banks as Bank 1, Bank 2, Bank 3, Bank 4, and Bank 5, respectively. The motivation for selection of these banks is to test the reliability of the proposed VaR models for the top UK banks in periods of distress. Therefore, our data covers the period from 31 December 2004 to 31 December 2015, covering the 2008 global financial crisis and the 2011 European financial crisis. All data are from DataStream.

In some literature the stability in financial systems is measured using a portfolio consisting of several banks (i.e., by considering the dependence among the banks), while other studies focus on individual banks. This paper considers both measures. Therefore, by using the stock prices for each bank, we calculate the log-return series and apply risk factor mappings to construct a simulated portfolio of returns for all banks as follows: Consider a portfolio consisting of *N* risk factors represented in vector form as *S*_*N*_ = (*s*_1*t*_, …, *s*_*Nt*_), the log-returns *r*_*t*_, are calculated as

$$\begin{array}{c} r_{t}=\left[\log\left(\frac{S_{1,t+\tau }}{S_{1,t}}\right),\ldots ,\log\left(\frac{S_{N,t+\tau }}{S_{N,t}}\right)\right]=\left(r_{1t},\ldots ,r_{Nt}\right). \end{array}$$ (38)

Let **Inv** be the total amount invested in the portfolio, *x*_*i*_ be the fraction of the total investment invested in stock *i*, *r*_*i*,*t*_ is the return of stock *i* at time *t*, then the weight applied to *r*_*i*,*t*_ is the fraction of the portfolio invested in stock *i* calculated as $w_{i}=\frac{x_{i}}{\text{Inv}}$. Since the stocks are all from banks of almost the same strength (i.e., the top five banks in the UK), we may assume equal weights. Therefore, the expected return on the portfolio at time *t* is given by

$$\begin{array}{c} {\bar{R}}_{p,t}=E\left(R_{p,t}\right)=\sum_{i=1}^{N}w_{i}E\left(r_{i,t}\right),\;\sum_{i=1}^{N}w_{i}=1, \end{array}$$ (39)

which is a weighted average of the return on the individual stocks in the portfolio.

**Fig 1:**
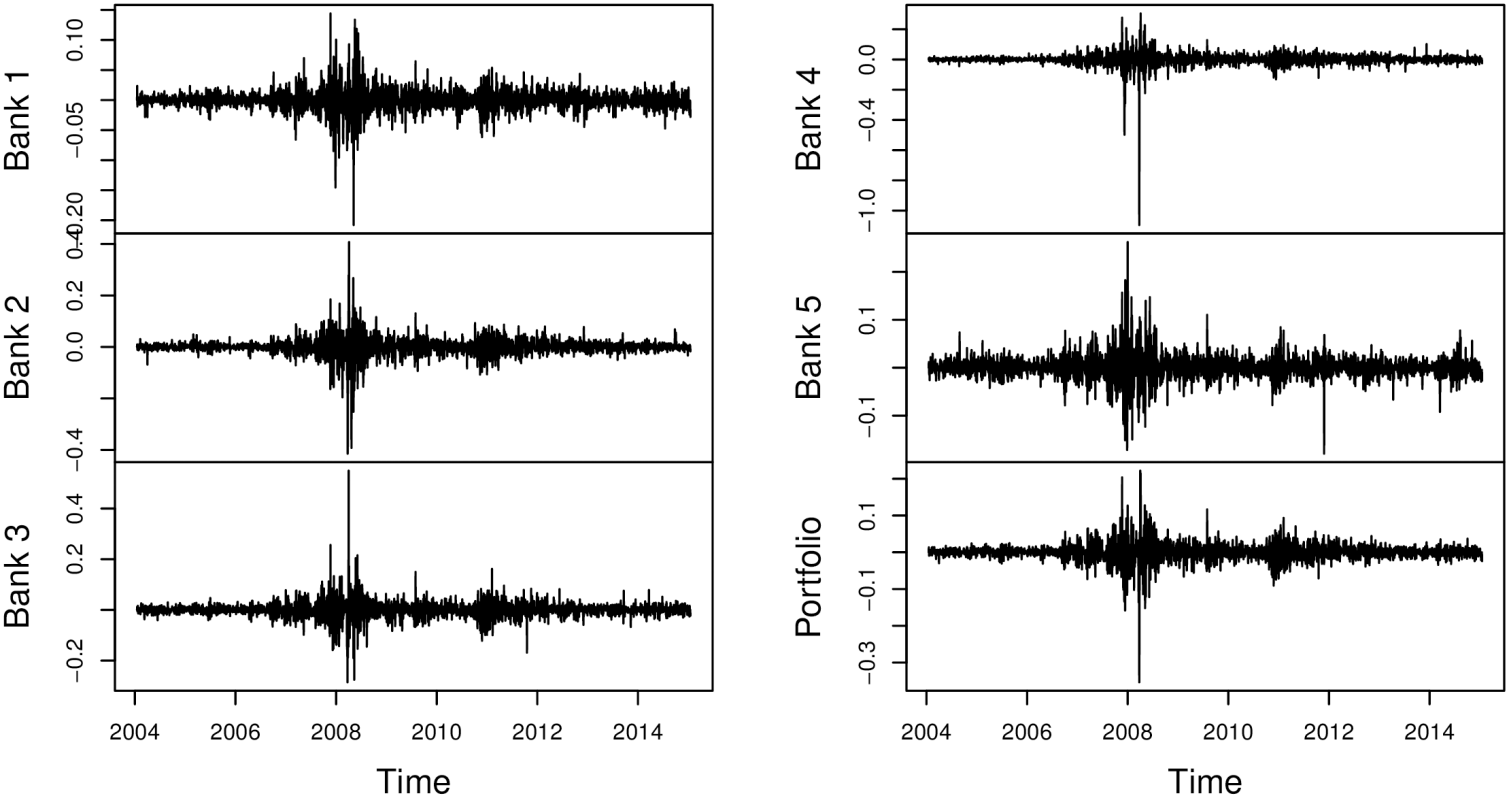
Time plots of the log-return series. Plots show the presence of volatility clustering in the log-return data.

Fig 1 shows time plots of the log-return series and the portfolio; this shows evidence of volatility clustering in the return series. From the figure, we can also see the effects of the 2008 global financial crisis and the 2011 European financial crisis.

Table 1 presents summary statistics of the data. We see from the table that the log-return series for each bank and the portfolio are far from being normally distributed as indicated by their high excess kurtosis and skewness. Furthermore, Jarque-Bera normality tests, Ljung-Box tests on the squared residuals $a_{i,t}^{2}$; where *a*_*i*,*t*_ = *r*_*i*,*t*_ − *μ*_*i*_ (*μ*_*i*_ being the unconditional mean), and a Lagrange multiplier tests for autoregressive conditional heteroscedasticity (ARCH LM test) on the residuals *a*_*i*,*t*_, as described in [55, 61, 62], are significant at 5% level.

**Table 1:** Summary statistics of daily log-returns and portfolio return series.

	Bank 1	Bank 2	Bank 3	Bank 4	Bank 5	Portfolio
Mean	-0.0001	-0.0004	-0.0003	-0.0010	-0.0001	-0.0004
Variance	0.0003	0.0011	0.0010	0.0015	0.0006	0.0006
Std. deviation	0.0171	0.0328	0.0321	0.0388	0.0244	0.0239
Skewness	-0.3367	-1.0549	1.4387	-8.4013	0.3161	-0.7549
Excess kurtosis	16.9080	37.2754	40.2179	235.5263	13.0850	28.6129

## Results

### Modelling the marginal distributions of volatility equations

As noted, the log-return series are leptokurtic and skewed. Thus, to capture the tail distribution and the dynamics of fluctuations in the time series data, we consider a single-state, *k* = 1 and two-state, *k* = {1, 2} Markov Switching GARCH specifications. The underlying volatility model is a GJR-GARCH(1,1) model with skewed Student’s-*t* distribution. Since we use just one variance specification (i.e., GJR-GARCH), the two-state Markov Switching GARCH is generated by setting the number of regimes in the conditional distribution to 2. For the single-state, the length of the variance specification is equal to the length of the conditional distribution, which is 1 (see [14]). Also note that the single-state Markov Switching GJR-GARCH(1,1) model corresponds to GJR-GARCH(1,1) model without regime change. Therefore, we simply refer to the single-state and two-state Markov Switching GJR-GARCH(1,1) models as GJR-GARCH(1,1) and MS-GJR-GARCH(1,1) models, respectively (see [14]). GARCH parameters are estimated using Bayesian statistics as follows: (i) We assign a prior distribution with initial hyperparameters and generate MCMC simulations of 40000 draws; (ii) if convergence is attained, we discard the first 20000 draws and select only the 10*th* draw from each chain such that auto-correlation between draws is reduced to almost zero. We merge the two chains together to obtain a sample data set of 2000 observations. (iii) If convergence is not attained, repeat (i) using parameter estimates from the previous draw as the hyperparameters to increase the chance of convergence. The mean value of each parameter with respect to its respective posterior distribution is the optimal parameter estimate of the Bayesian GJR-GARCH(1,1) and Bayesian MS-GJR-GARCH(1,1) models with skewed Student’s-*t* distributions. Estimation results are presented in Tables 2 and 3 with standard errors in parenthesis. For MS-GJR-GARCH(1,1) model, the degrees of freedom parameter, *ν* is fixed across the regimes.

**Table 2:** Parameter estimates following Bayesian GJR-GARCH(1,1) model with skewed Student’s-*t* distribution.

	*α*_0_	*α*_1_	*α*_2_	*β*_1_	*ν*	*γ*
Bank 1	7.0531e-06(0.0000)	0.0508(0.0010)	0.1001(0.0000)	0.8488(0.0002)	5.8153 (0.0114)	1.0067(0.005)
Bank 2	1.4764e-6(0.0000)	0.0509(0.0000)	0.1001(0.0000)	0.8570(0.0001)	6.4085 (0.0133)	1.0014 (0.0005)
Bank 3	7.0777e-06(0.0000)	0.0511(0.0000)	0.1001(0.0000)	0.8716(0.0001)	6.1691 (0.0118)	1.0009 (0.0005)
Bank 4	9.4281e-06(0.0000)	0.0511(0.0000)	0.1002(0.0000)	0.8688(0.0001)	5.9166 (0.0111)	1.0160 (0.0005)
Bank 5	2.0683e-05(0.0000)	0.0508(0.0000)	0.1002(0.0000)	0.8321(0.0002)	6.3657 (0.0138)	1.0266 (0.0005)
Portfolio	5.4112e-06(0.0000)	0.0510(0.0000)	0.1002(0.0000)	0.8670(0.0001)	9.4379 (0.0298)	0.9936 (0.0005)

Note: Standard errors in parentheses.

**Table 3:** Parameter estimates for two-state MS-GJR-GARCH(1,1) model with skewed Student’s-*t* distribution.

	*k* = 1					
	*α*_0___1_	*α*_1___1_	*α*_2___1_	*β*_1___1_	*ν*	*γ*__1_
Bank 1	2.9335e-07(0.0000)	0.0270 (0.0010)	0.0121 (0.0004)	0.9612 (0.0005)	6.2679 (0.0159)	1.0380 (0.0009)
Bank 2	1.9132e-06(0.0000)	0.0302 (0.0004)	0.0811 (0.0014)	0.9208 (0.0011)	7.9120 (0.0270)	1.0115 (0.0010)
Bank 3	2.7159e-07(0.0000)	0.0109 (0.0002)	0.0253 (0.0003)	0.9729 (0.0002)	5.6388 (0.0142)	0.9516 (0.0007)
Bank 4	1.0034e-07(0.0000)	0.0367 (0.0002)	0.0030 (0.0001)	0.9595 (0.0002)	7.4127 (0.0220)	1.0146 (0.0009)
Bank 5	3.8283e-06(0.0000)	0.0338 (0.0008)	0.0932 (0.0032)	0.9083 (0.0027)	7.2792 (0.0186)	1.0462 (0.0011)
Portfolio	1.5441e-05(0.0000)	0.0341 (0.0004)	0.1694 (0.0040)	0.8665 (0.0025)	14.1506 (0.0621)	0.9954 (0.0013)
	*k* = 2					
	*α*_0___2_	*α*_1___2_	*α*_2___2_	*β*_1___2_	*ν*	*γ*__2_
Bank 1	1.0589e-05(0.0000)	0.0412 (0.0005)	0.1568 (0.0011)	0.8566 (0.0007)	6.2679 (0.0159)	0.9496 (0.0012)
Bank 2	1.8650e-05(0.0000)	0.0056 (0.0002)	0.2322 (0.0027)	0.8586 (0.0015)	7.9120 (0.0270)	0.9782 (0.0018)
Bank 3	1.5749e-05(0.0000)	0.0558 (0.0004)	0.0776 (0.0014)	0.9045 (0.0006)	5.6388 (0.0142)	1.1826 (0.0026)
Bank 4	6.8672e-05(0.0000)	0.0683 (0.0013)	0.8202 (0.0043)	0.4771 (0.0022)	7.4127 (0.0220)	1.0138 (0.0023)
Bank 5	1.8827e-05(0.0000)	0.1016 (0.0011)	0.3991 (0.0040)	0.6778 (0.0028)	7.2792 (0.0186)	1.0085 (0.0015)
Portfolio	2.2462e-06(0.0000)	0.0334 (0.0004)	0.2052 (0.0040)	0.8543 (0.0023)	14.1506 (0.0298)	0.9936 (0.0005)

Note: Standard errors in parentheses. Degrees of freedom parameter, *ν* is fixed across the regimes.

Applying Eq (2), we then obtain a matrix Σ, which consists of the filtered marginal standardised residuals, ${\left\{\epsilon_{i,t}\right\}}_{t=1}^{T}$, of the overall process for the MS-GJR-GARCH(1,1) model and GJR-GARCH(1,1) model. That is

$$\begin{array}{c} \Sigma_{i,t}=\left(r_{i,t}\right)\left(h_{\Delta_{i,t},i,t}^{-\frac{1}{2}}\right),\;i=1,\ldots ,N;\;t=1,\ldots ,T. \end{array}$$ (40)

The ARCH LM test and Ljung-Box test on the standardised residuals and standardised squared residuals, respectively, for lags 5 and 10 are presented in Table 4. For the GJR-GARCH(1,1) model, there still exist some serial correlation in the standardised residuals of bank 4. For MS-GJR-GARCH(1,1) model, there is no evidence of an ARCH effect or serial correlations in the standardised residuals.

**Table 4:** ARCH LM test on the standardised residuals and Ljung-Box test on the standardised squared residuals for *k* = 1. The null hypothesis of no ARCH effect or serial correlation is rejected at 5% significant level for Bank 4.

*k* = 1	ARCH LM test						Ljung-Box test				
	Bank 1	Bank 2	Bank 3	Bank 4	Bank 5		Bank 1	Bank 2	Bank 3	Bank 4	Bank 5
LM(5)	2.21	2.35	2.96	17.78	4.01	Q(5)	2.24	2.35	2.95	17.65	4.11
*p*-value	0.820	0.800	0.706	0.003	0.548	*p*-value	0.815	0.799	0.707	0.003	0.534
LM(10)	10.26	4.13	7.04	18.62	6.79	Q(10)	9.92	4.14	7.17	18.57	6.89
*p*-value	0.820	0.942	0.722	0.045	0.745	*p*-value	0.447	0.941	0.710	0.046	0.736
*k* = {1, 2}	ARCH LM test						Ljung-Box test				
	Bank 1	Bank 2	Bank 3	Bank 4	Bank 5		Bank 1	Bank 2	Bank 3	Bank 4	Bank 5
LM(5)	2.164	2.29	8.74	5.31	3.06	Q(5)	2.13	2.22	8.60	5.30	10.56
*p*-value	0.826	0.807	0.120	0.379	0.690	*p*-value	0.831	0.818	0.126	0.380	0.061
LM(10)	6.01	3.75	13.39	5.83	5.30	Q(10)	5.98	3.70	13.27	5.88	12.71
*p*-value	0.815	0.958	0.203	0.829	0.870	*p*-value	0.817	0.960	0.209	0.826	0.240

Note: For *k* = 1, we have a GJR-GARCH(1,1) model, and for *k* = {1, 2}, we have a MS-GJR-GARCH(1,1) model.

### Modelling dependence with copulas

We model the dependence structure among the stock returns using copula functions. Copula parameters are estimated by the canonical maximum likelihood (CML) method [41]. This entails the use of pseudo-observations of the standardised residuals to estimate the marginals. We then estimate the copula parameters by inversion of Kendall’s *τ*, which is one of the most commonly used invariant measures and has been proven to provide more efficient ways of estimating correlations [63, 64]. The copula that fits the data best is selected by maximum likelihood estimation (MLE) method by maximising the likelihood function

$$\begin{array}{c} {\hat{\Psi }}_{2}=ArgMax_{\Psi_{2}}\sum_{t=1}^{T}\ln c({\hat{F}}_{1}\left(X_{1t}\right),\ldots ,{\hat{F}}_{n}\left(X_{nt}\right);\Psi_{2}), \end{array}$$ (41)

where ${\hat{\Psi }}_{2}$ are estimates of the copula parameters. The estimated copula parameters are reported in Table 5, along with their Akaike information criterion (AIC) values. For both models, Frank and Student’s-*t* copulas are selected from each copula family based on the highest MLE values. From Table 5, the same copula types have been selected based on the AIC values (the copula with the smallest AIC value is preferred). Note that Gaussian copula gives a higher MLE value compared to the Archimedean copulas but also higher AIC value. Table 6 shows the Kendall’s *τ* for Gaussian and Student’s-*t* copula parameter estimates. Thus, the Gaussian copula is not a good fit for the data. The analysis continues based on the selected copulas. Next, we specify the desired marginal distributions, which we set to Student’s-*t* distribution, and using the estimated copula parameters, we generate 10000 simulations to obtain a new matrix of marginal standardised residuals

$$\begin{array}{c} \hat{\Sigma }=\left\{\zeta_{i,j}\right\},\;j=1,\ldots ,T,\;i=1,\ldots ,N, \end{array}$$ (42)

which is free from assumptions of normality and linear correlations. To confirm this, we employ a multivariate ARCH test based on the Ljung-Box test statistics

$$\begin{array}{c} Q_{k}\left(m\right)=T^{2}\sum_{i=1}^{m}\frac{1}{T-i}b_{i}^{\prime }\left({\hat{\rho }}_{0}^{-1}⊗{\hat{\rho }}_{0}^{-1}\right)b_{i}\approx \chi_{k^{2}}^{2}\left(m\right), \end{array}$$ (43)

and its modification $Q_{k}^{r}\left(m\right)$, known as a robust test, on the log returns at 5% significance level, where *m* is the number of lags of cross-correlation matrices used in the tests, *k* is the dimension of *r*_*i*,*t*_, *T* is the sample size, $b_{i}=\text{vec}\left({\hat{\rho }}_{i}^{\prime }\right)$ with ${\hat{\rho }}_{j}$ being the lag-*j* cross-correlation matrix of $r_{i,t}^{2}$. The modification $Q_{k}^{r}\left(m\right)$ involves discarding those observations from the return series whose corresponding standardised residuals exceed 95*th* quantile in order to reduce the effect of heavy tails. The motivation for $Q_{k}^{r}\left(m\right)$ test is that *Q*_*k*_(*m*) may fare poorly in finite samples when the residuals of the time series, *r*_*i*,*t*_, have heavy tails [45]. The tests show no evidence of conditional heteroscedasticity lags *m* = 10; Table 7.

**Table 5:** Copula parameter estimates are based on inversion of Kendall’s *τ* following CML estimation method.

GJR-GARCH(1,1)	Archimedean copulas			Elliptical copulas	
	Gumbel	Clayton	Frank	Gaussian	Student’s-*t*
Kendall’s *τ*	1.782 (0.023)	1.563 (0.046)	4.697 (0.042)	*ρ*_*G*_ = *ρ*_*τ*_(*ρ*_*SE*_)	*ρ*_*t*_ = *ρ*_*τ*_(*ρ*_*SE*_)
MLE	3226	2745	**3250**	3846	**4108**
AIC	-14.158	-13.835	**-14.173**	3.491	**3.359**
MS-GJR-GARCH(1,1)					
	Gumbel	Clayton	Frank	Gaussian	Student’s-*t*
Kendall’s *τ*	1.773 (0.023)	1.546 (0.046)	4.657 (0.041)	*ρ*_*G*_ = *ρ*_*τ*_(*ρ*_*SE*_)	*ρ*_*t*_ = *ρ*_*τ*_(*ρ*_*SE*_)
MLE	3163	2705	**3206**	3773	**4013**
AIC	-14.119	-13.806	**-14.146**	3.529	**3.405**

Note: Standard errors in parentheses. The best copula for modeling dependence among the risk factors is that with the highest MLE value or smallest AIC value (in bold).

**Table 6:** Kendall’s *τ*; *ρ*_*τ*_(*ρ*_*SE*_) for Gaussian and Student’s-*t* copula parameter estimates.

		Bank 1	Bank 2	Bank 3	Bank 4	Bank 5
GJR-GARCH(1,1)	Bank 1	1				
	Bank 2	0.6230 (0.013)	1			
	Bank 3	0.5521 (0.015)	0.7054 (0.011)	1		
	Bank 4	0.5741 (0.014)	0.7262 (0.011)	0.7176 (0.011)	1	
	Bank 5	0.6383 (0.013)	0.6027 (0.014)	0.5437 (0.015)	0.5460 (0.015)	1
MS-GJR-GARCH(1,1)	Bank 1	1				
	Bank 2	0.6257 (0.013)	1			
	Bank 3	0.5544 (0.015)	0.7074 (0.011)	1		
	Bank 4	0.5779 (0.015)	0.7282 (0.011)	0.7225 (0.011)	1	
	Bank 5	0.6437 (0.012)	0.6075 (0.014)	0.5439 (0.015)	0.5483 (0.015)	1

Note: Standard errors in parentheses.

**Table 7:** Multivariate ARCH test on {*ζ*_*i*,*j*_} shows no evidence of conditional heteroscedasticity.

	GJR-GARCH(1,1)		MS-GJR-GARCH(1,1)	
Frank copula	*Q*_*k*_(10) = 11.413	$Q_{k}^{r}\left(10\right)$ = 267.925	*Q*_*k*_(10) = 5.288	$Q_{k}^{r}\left(10\right)$ = 245.072
	*p*-value = 0.326	*p*-value = 0.208	*p*-value = 0.871	*p*-value = 0.576
t-copula	*Q*_*k*_(10) = 5.507	$Q_{k}^{r}\left(10\right)$ = 235.133	*Q*_*k*_(10) = 2.554	$Q_{k}^{r}\left(10\right)$ = 249.171
	*p*-value = 0.855	*p*-value = 0.742	*p*-value = 0.990	*p*-value = 0.503

**Fig 2:**
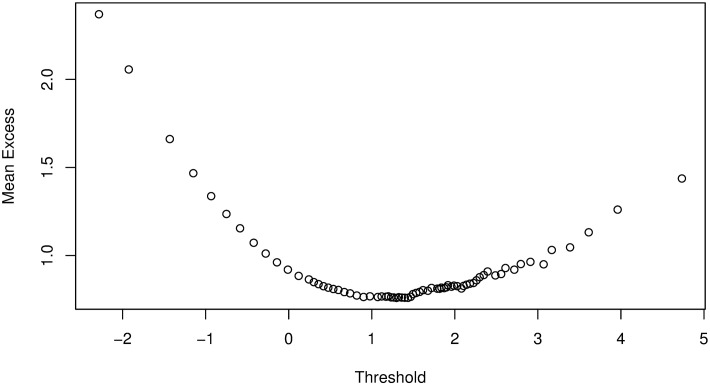
Mean excess function plot for Bank 1 following analysis with Bayesian GJR-GARCH(1,1) Student’s-*t* copula model.

**Fig 3:**
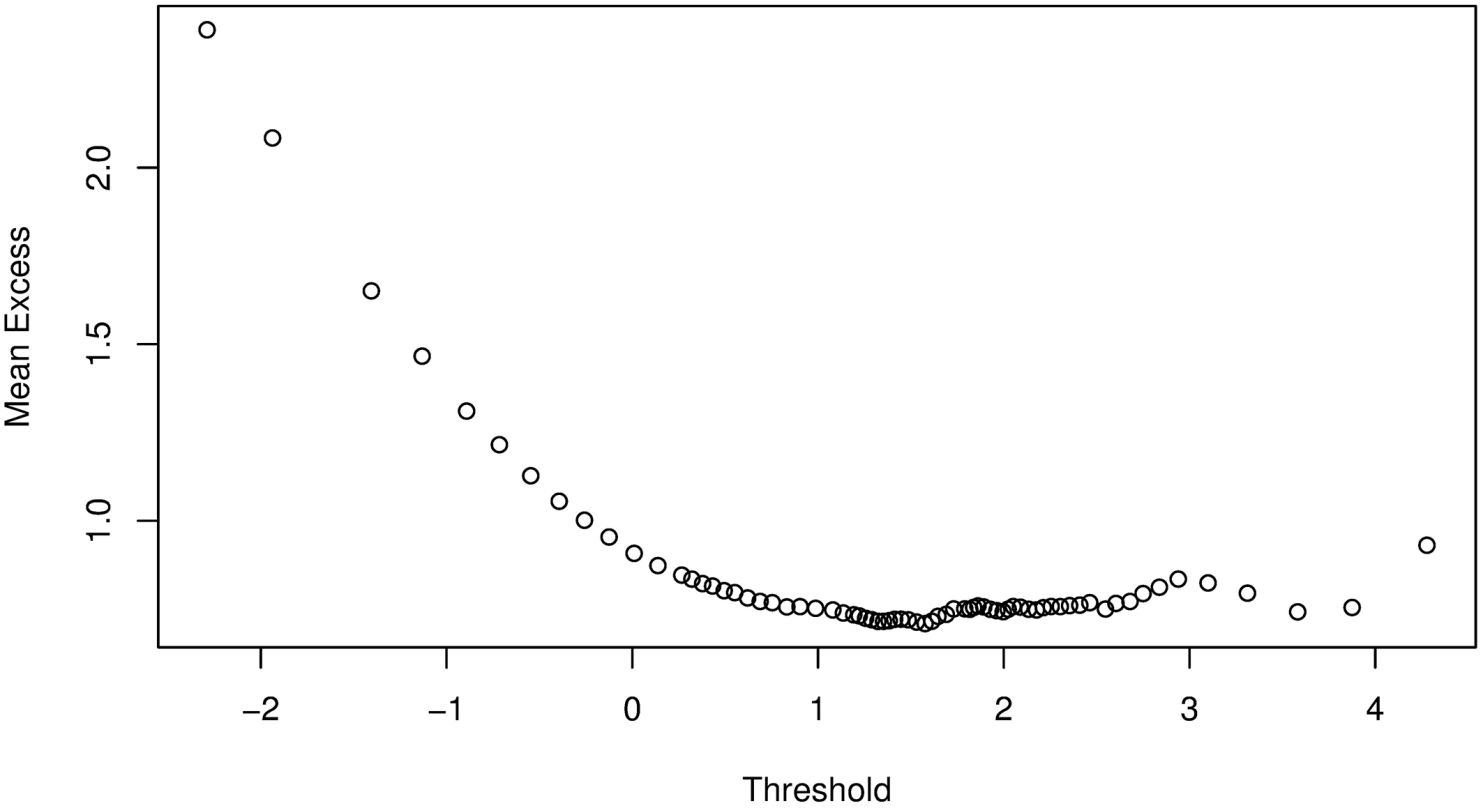
Mean excess function plot for Bank 1 following analysis with Bayesian GJR-GARCH(1,1) Frank copula model.

We follow the approach by [9] and apply the POT method of EVT to each of the marginal distributions of {*ζ*_*i*,*t*_} (i.e., Eq (42)) to obtain the *q*^*th*^ quantile, *VaR*(*Z*)_*q*_ of the noise variables for VaR estimation. Let {*χ*_*i*,*τ*_} be the negative variables of the marginal distributions of {*ζ*_*i*,*t*_} such that {*χ*_*i*,*τ*_} ⊆ {*ζ*_*i*,*t*_}. Then, from the ordered sample of {*χ*_*i*,*τ*_}, we calculate and plot the mean excess function to help identify the threshold. As an example, Figs 2 and 3 are mean excess function plots for Bank 1 following the Bayesian GJR-GARCH(1,1) Student’s-*t* and Frank copula models. The plots suggest us to select threshold values of about 1.3 and 1.4 for Figs 2 and 3, respectively, which are the lowest points on the graphs above which the graph appears to be approximately linear. However, if we select these points as the threshold values, we will have 1402 exceedances for Fig 2 and 1039 exceedances for Fig 3, which are too many compared to the size of the data (i.e., *T* = 10000). The number of exceedances thus lie towards the body of the data, which will inevitably result in a poor approximation of the GPD parameters and hence lead to inaccuracies in the VaR estimate. In addition, the threshold selection method is very subjective and will be different from one analyst to the other based on their preferences.

**Fig 4:**
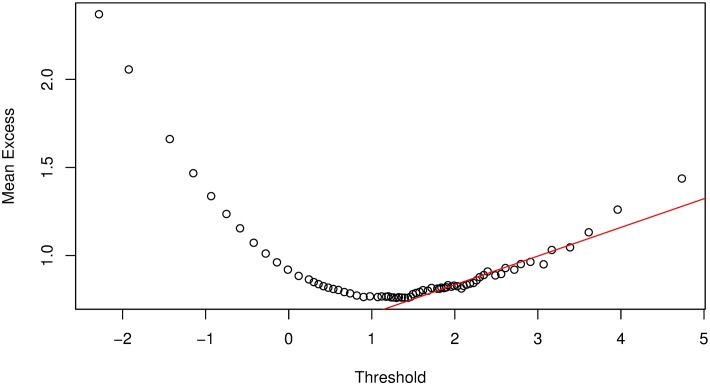
Mean excess function plot demonstrating the *hybrid* method for threshold selection.

We propose an extension to the mean excess plot for threshold selection; the *hybrid* method. That is, from the mean excess plot, we identify the lowest point, making the graph appears approximately linear, a point *ϑ*_0_. We then insert a tangent line from *ϑ*_0_ through the rest of the points *ϑ*_*i*_, where *ϑ*_*i*_ > *ϑ*_0_; see Fig 4. Since the tangent to a linear curve is the tangent itself and the mean excess function is a linear function of the threshold, we take an average of the set of points that touches the tangent line as the threshold value, a point ϑ*. This point ϑ* will lead to a better approximation of VaR estimates than *ϑ*_0_ because the inference is restricted to the left tail. Apart from better approximation of VaR estimates, this method significantly reduces the probability of having different VaR estimates for the same data and also the probability of selecting a very low or very high threshold value. Let *ϑ*_*i*_ = *ϑ*_1_, …, *ϑ*_ℏ_ be a set of points that touches the tangent line, then we obtain the value of ϑ* as

$$\begin{array}{c} \vartheta^{*}=\frac{1}{\hbar }\sum_{i=1}^{\hbar }\vartheta_{i},\;\vartheta_{i}\geq \vartheta_{0}, \end{array}$$ (44)

where ℏ is the number of points in the set.

**Fig 5:**
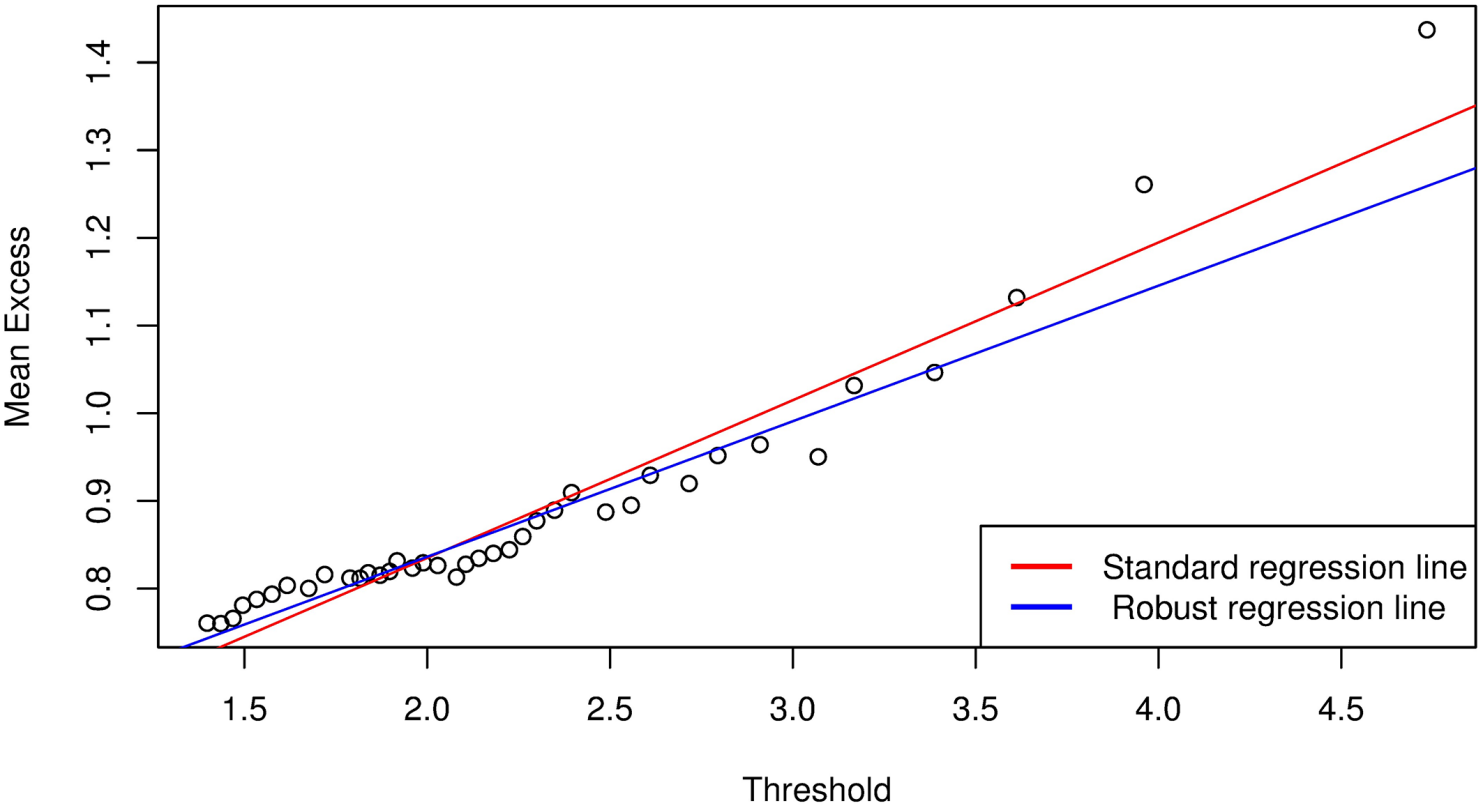
Mean excess function plot following analysis with Bayesian GJR-GARCH(1,1) Student’s-*t* copula model for the number of exceedances above *ϑ*_0_. A reliable threshold is calculated by taking an average of the set of points that touches the robust regression line. The standard regression line is affected by outliers in the left tail.

**Fig 6:**
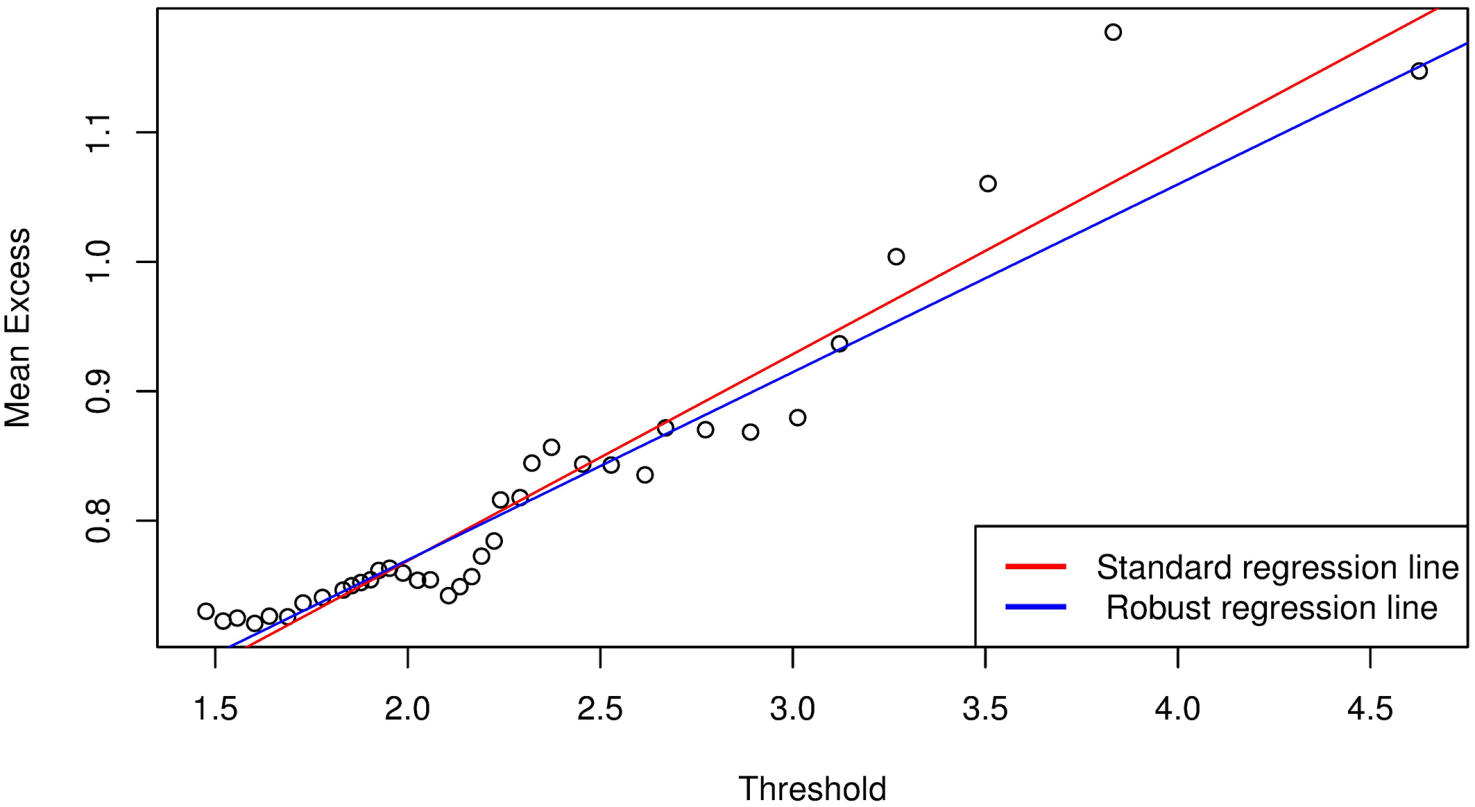
Mean excess function plot following analysis with Bayesian GJR-GARCH(1,1) Frank copula model for the number of exceedances above *ϑ*_0_.

It can be seen in Fig 4 that the points touching the tangent line, i.e., *ϑ*_0_, are too compact and might lead us to miss some important points. A better way for selecting these points is by fitting a regression line

$$\begin{array}{c} \hat{y}=b_{0}+b_{1}x, \end{array}$$ (45)

which is based on the least square method to the points ${\left\{\vartheta_{i}\right\}}_{i=1}^{\hbar }$, where $\hat{y}$ is the estimate of the dependent variable, and *x* is the independent variable with intercept *b*_0_ and slope *b*_1_. In the presence of heteroscedasticity and outliers, it may be advantageous to consider fitting a robust regression line. Robust regression methods are not influenced by outliers, and are also very useful when there are problems with heteroscedasticity in the data set. This method is demonstrated for Bank 1 in Figs 5 and 6, which illustrates a comparison between simple linear and robust regression methods. It can be seen that the regression lines for standard regression models are affected by outliers in the left tails of the mean excess plots, hence a robust regression model is more reliable.

Following the above analysis, we obtained a threshold value of 2.2448 and 333 exceedances for Fig 5, and 2.6476 and 176 exceedances for Fig 6. The analysis are restricted to the tails and the data is sufficient to allow for reasonable statistical inferences with EVT. Tables 8 and 9 presents the POT parameter estimates and the forecast VaR estimates. The portfolio VaR estimates, ${VaR}_{q}^{p}\left(Z\right)$, based on the individual bank’s VaR estimates and confidence level are also reported. ${VaR}_{q}^{p}\left(Z\right)$ is computed using the risk formula

$$\begin{array}{c} {VaR}_{q}^{p}\left(Z\right)={\left(\sum_{i=1}^{N}w_{i}^{2}{VaR}_{q,i}^{2}\left(Z\right)+2w_{i}w_{j}\sum_{i<j}^{N}\rho_{ij}{VaR}_{q,i}\left(Z\right){VaR}_{q,j}\left(Z\right)\right)}^{\frac{1}{2}},\;\sum_{i=1}^{N}w_{i}=1, \end{array}$$ (46)

where *ρ*_*i*,*j*_ is the Pearson cross-correlation coefficient between the returns of the *i*th and *j*th stocks. As noted, the overall risk measures are quite stable for both models and different thresholds indicating that the model has effectively captured the dynamics of fluctuations in the left tails of the return distributions. This claim may be validated through back-testing the model. We can also see the effect of diversification on the risk of the individual banks on the portfolio VaR. Employing Eq (46), the one step ahead VaR is then calculated as

$$\begin{array}{c} {VaR}_{q,t}^{p}={VaR}_{q}^{p}\left(Z\right){\hat{h}}_{\Delta_{t},t+1}^{\frac{1}{2}}. \end{array}$$ (47)

Note that ${\hat{h}}_{\Delta_{t},t+1}^{\frac{1}{2}}$ is the one-step-ahead conditional volatility forecast of the overall conditional variance for the portfolio at time *t* + 1 for state *k*, Δ_*t*_ is a Markov chain as defined in Eqs (1) and (2), but for ${\bar{R}}_{p}$ is by Eq (39). That is, ${\bar{R}}_{p,t}|\left(\Delta_{t}=k,\Omega_{t-1}\right)$ and the parameters are sampled from the posterior distribution using MH algorithm.

**Table 8:** POT parameter estimates, *VaR*_*q*_(*Z*) and ${VaR}_{q}^{p}\left(Z\right)$ following Bayesian GJR-GARCH(1,1) Frank and Student’s-*t* copula-EVT models.

		Parameters						*VaR*_*q*_(*Z*)	
		*ξ*	*ψ*(ϑ*)	ϑ*	*N*_ϑ*_	*μ*	*σ*	99%	95%
Student’s-*t* copula:	Bank 1	0.1660	0.7143	2.2448	333	0.3881	0.4061	3.1958	1.9640
	Bank 2	0.2239	0.6479	2.4624	218	0.7974	0.2750	3.0141	1.9716
	Bank 3	0.1838	0.6353	2.2321	356	0.6481	0.3441	3.1407	2.0229
	Bank 4	0.1273	0.7301	2.4687	271	0.3567	0.4612	3.2448	2.0385
	Bank 5	0.1465	0.7135	2.3293	287	0.3538	0.4242	3.1428	1.9489
${VaR}_{q}^{p}\left(Z\right)$								2.5862	1.6282
Frank copula:	Bank 1	0.1019	0.7239	2.6476	176	0.2503	0.4796	3.0688	1.9305
	Bank 2	0.0497	0.7489	2.4331	235	-0.1297	0.6216	3.0867	1.8781
	Bank 3	0.0390	0.7892	2.6040	223	-0.1855	0.6804	3.2469	1.9767
	Bank 4	0.2073	0.6862	2.5407	217	0.7266	0.3102	3.1174	2.0147
	Bank 5	0.1062	0.6892	3.1337	105	0.6440	0.4249	3.1674	2.1425
${VaR}_{q}^{p}\left(Z\right)$								2.5410	1.6459

Note: Estimations for a time horizon of 1 day at *q* = (99%, 95%) confidence level. The risk measures are quite stable for different thresholds and copula functions indicating that the VaR models have successfully capture the dynamics of fluctuations in the left tails.

**Table 9:** POT parameter estimates, *VaR*_*q*_(*Z*) and ${VaR}_{q}^{p}\left(Z\right)$ following Bayesian MS-GJR-GARCH(1,1) Frank and Student’s-*t* copula-EVT models.

		Parameters						*VaR*_*q*_(*Z*)	
		*ξ*	*ψ*(ϑ*)	ϑ*	*N*_ϑ*_	*μ*	*σ*	99%	95%
Student’s-*t* copula:	Bank 1	0.0194	0.8795	1.8425	565	-0.4893	0.8343	3.1862	1.9389
	Bank 2	0.0239	0.8003	1.9903	409	-0.3040	0.7455	2.9471	1.8512
	Bank 3	0.0462	0.7642	2.0585	453	-0.1450	0.6624	3.2542	1.9832
	Bank 4	0.0686	0.6675	2.3155	260	0.7943	0.5632	3.0091	1.8140
	Bank 5	0.0038	0.6891	1.9164	493	-0.1460	0.6814	3.0191	1.9067
${VaR}_{q}^{p}\left(Z\right)$								2.5127	1.5471
Frank copula:	Bank 1	0.0868	0.8295	2.5454	201	-0.2030	0.5910	3.1457	1.9804
	Bank 2	0.0970	0.7780	1.9443	440	-0.7856	0.5132	2.9769	1.8480
	Bank 3	0.0819	0.7132	2.1074	358	0.0287	0.5430	3.0662	1.8724
	Bank 4	0.0806	0.6030	2.5029	200	0.4796	0.4399	2.9327	1.9703
	Bank 5	0.0924	0.6688	2.3509	230	0.2210	0.4719	2.9300	1.8498
${VaR}_{q}^{p}\left(Z\right)$								2.4535	1.5513

Note: Estimations for a time horizon of 1 day at *q* = (99%, 95%) confidence level. The risk measures are quite stable for different thresholds and copula functions indicating that the VaR models have successfully capture the dynamics of fluctuations in the left tails.

**Fig 7:**
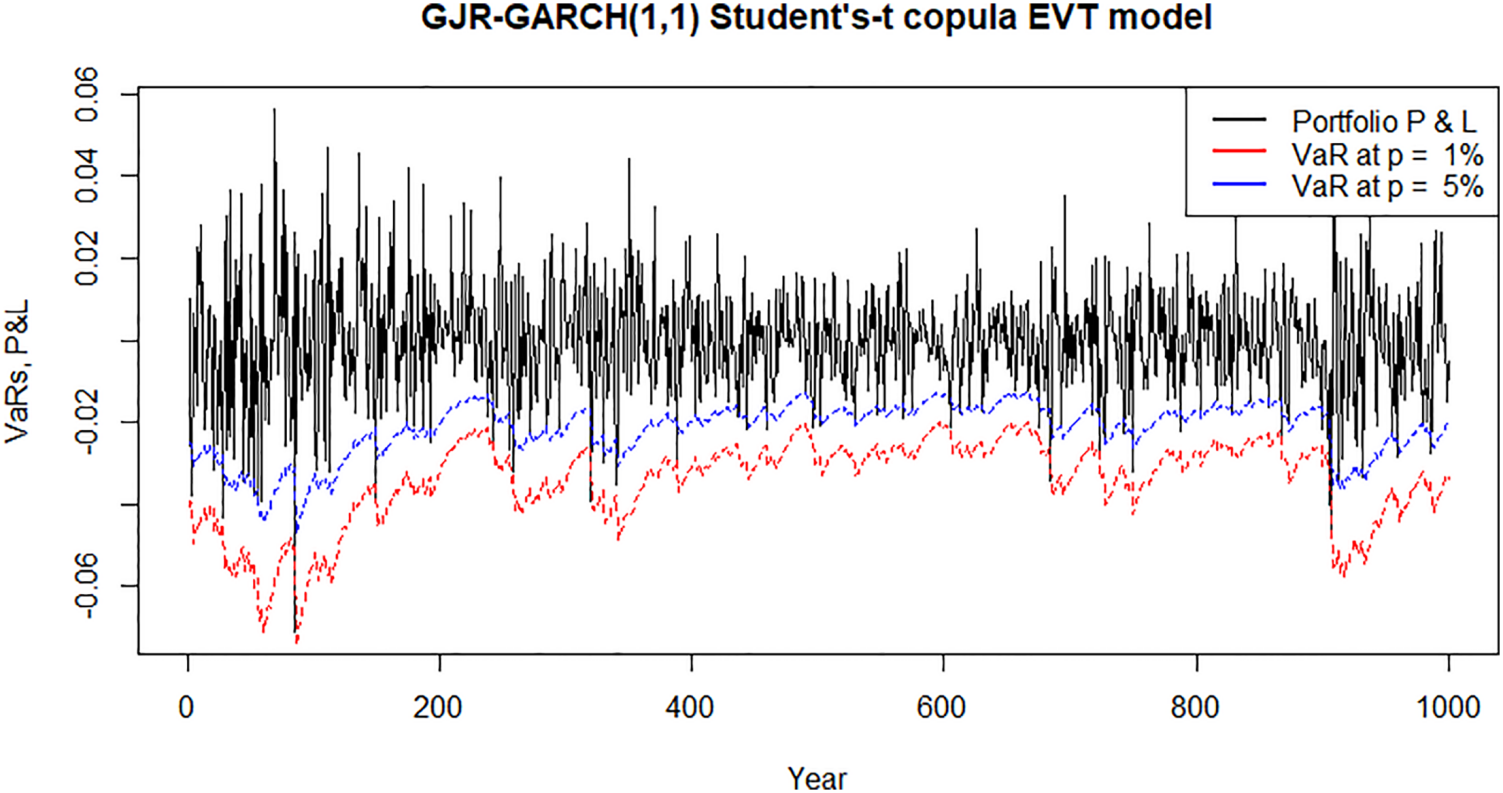
Forecasts daily VaR estimates and daily profit and loss (P&L) plots for an investment in a portfolio consisting of all banks following Bayesian GJR-GARCH(1,1) Student’s-*t* copula EVT VaR model.

**Fig 8:**
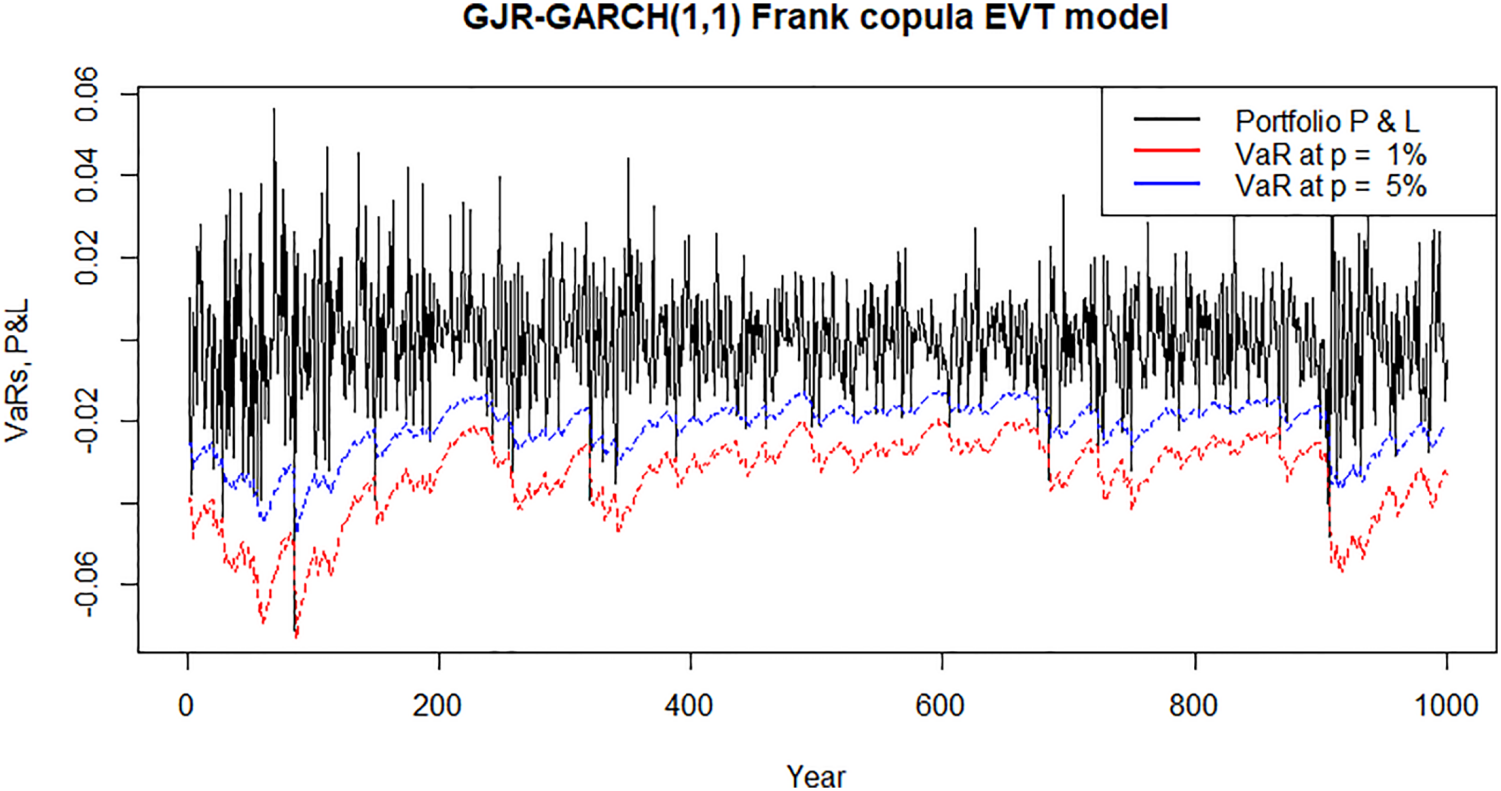
Forecasts daily VaR estimates and daily profit and loss (P&L) plots for an investment in a portfolio consisting of all banks following Bayesian GJR-GARCH(1,1) Frank copula EVT VaR model.

**Fig 9:**
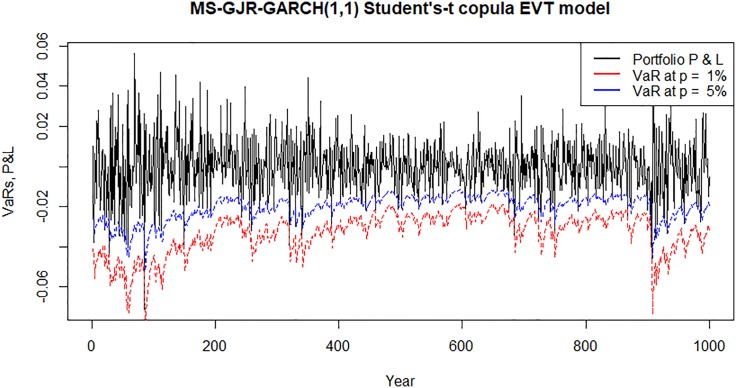
Forecasts daily VaR estimates and daily profit and loss (P&L) plots for an investment in a portfolio consisting of all banks following Bayesian MS-GJR-GARCH(1,1) Student’s-*t* copula EVT VaR model.

**Fig 10:**
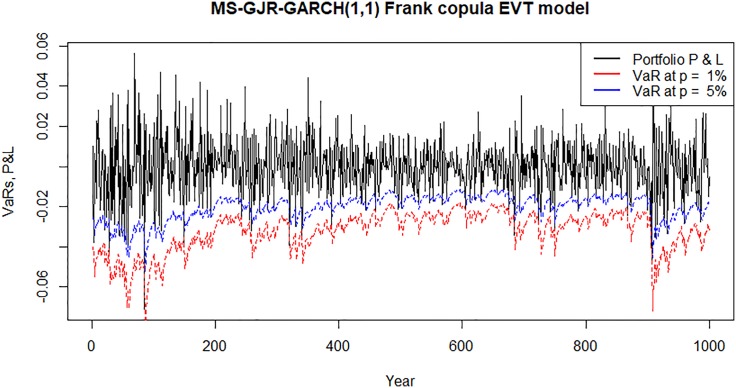
Forecasts daily VaR estimates and daily profit and loss (P&L) plots for an investment in a portfolio consisting of all banks following Bayesian MS-GJR-GARCH(1,1) Frank copula EVT VaR model.

Figs 7–10 show time plots of profit and loss (P&L) of the portfolio return series and forecasts portfolio VaR estimates at 99% and 95% confidence levels. A visual observation of the plots suggests that the VaR models perform quite well in capturing the dynamics in the portfolio return series.

### Model checking

The reliability of the VaR model is often tested by performing back-testing. This involves comparing the estimated VaRs for a given time horizon and observation period to the subsequent returns and recording the number of days in which the loss on the portfolio exceeds VaR. The number of days *T*_1_ in which the loss on the portfolio exceeds VaR is recorded as the number of exceptions or failures. Too many exceptions imply that the VaR model underestimates the level of risk, and too few exceptions imply the model overestimates risk. For the VaR model to be accepted as a reliable risk measure, the number of exceptions produced for any given observation period should satisfy the unconditional coverage (UC) and independent (IND) properties. We define an indicator function on the exceptions at time *t* as

$$\begin{array}{c} I_{t}\left(1-q\right)=I_{\left\{L_{t}>{VaR}_{q,t}^{p}\right\}}=\left\{ \begin{array}{c} 1,\;\text{if}\;L_{t}>{VaR}_{q,t}^{p}\\ 0,\;\text{otherwise}, \end{array}\right. \end{array}$$ (48)

where the indicator function equal to 1 when the loss on the portfolio *L*_*t*_ exceed ${VaR}_{q,t}^{p}$, and 0 if otherwise; note that *q* is the choice of confidence level. For the UC property, $\mathrm{Pr}\left[I_{t}\left(1-q\right)=1\right]\approx 1-q,\forall_{t}$; i.e., the number of exceptions should be reasonably close to *T*_*w*_(1 − *q*)%, depending on the choice of *q*, and should follow a binomial distribution. *T*_*w*_ is the size of the window over which back-testing is conducted. For the IND property, the exceptions produced on day *t* − 1 should be independent of exceptions produced on day *t* and evenly spread over time.

In this study, we use several back-testing methods to test the accuracy of the proposed VaR models. The most common are the Kupiec’s proportion of failures (POF) test for the UC [65], Christoffersen’s test for the UC and IND [66], Engle and Manganelli’s Dynamic Quantile (*DQ*) test [67], and Santos and Alves’ new class of independence test [68]. We also consider the Basel *traffic light* test proposed by the Basel Committee on Banking and Supervision (BCBS) [69].

Kupiec defined an approximate 95% confidence region whereby the number of exceptions produced by the VaR model must lie within this interval for it to be considered a reliable risk measurement model. The test is based on the likelihood ratio

$$\begin{array}{c} LR_{UC}=-2\ln\frac{q^{T_{0}}{\left(1-q\right)}^{T_{1}}}{{\left(1-\frac{T_{1}}{T_{w}}\right)}^{T_{0}}{\left(\frac{T_{1}}{T_{w}}\right)}^{T_{1}}}\approx \chi_{1}^{2}, \end{array}$$ (49)

where *T*_0_ = *T*_*w*_ − *T*_1_. Under the UC, the null hypothesis for *LR*_*POF*_ is $H_{0}:\text{E}\left[I_{t}\left(1-q\right)\right]=\frac{T_{1}}{T_{w}}=1-q$ against $H_{a}:\text{E}\left[I_{t}\left(1-q\right)\right]=\frac{T_{1}}{T_{w}}\neq \left(1-q\right)$. The VaR model is rejected if $LR_{POF}>\chi_{1}^{2}=3.841$.

A study by [66] extended Kupiec’s POF test to test the independence of conditional coverage. Under the null hypothesis that the number of exceptions produced are independent and evenly spread over time, *π*_01_ = *π*_11_ = *π* with likelihood ratio

$$\begin{array}{c} LR_{IND}=-2\ln\frac{{\left(1-\pi \right)}^{\left(T_{00}+T_{10}\right)}\pi^{\left(T_{01}+T_{11}\right)}}{{\left(1-\pi_{01}\right)}^{T_{00}}\pi_{01}^{T_{01}}{\left(1-\pi_{11}\right)}^{T_{10}}\pi_{11}^{T_{11}}}\approx \chi_{1}^{2}, \end{array}$$ (50)

where *T*_*ij*_, with *i*, *j* = 0(*noviolation*), 1(*violation*), is the number of observed events with the *j*^*th*^ event following *i*^*th*^, and *π*_01_, *π*_01_ and *π* are estimates of the probabilities of *T*_*i*,*j*_ [70]. The model is rejected for the independent property if $LR_{IND}>\chi_{1}^{2}=3.841$. Christoffersen conditional coverage test is a joint test of Kupiec’s POF test and the IND that test both properties of UC and IND instantaneously. The conditional coverage test has a likelihood ratio

$$\begin{array}{c} LR_{CC}=LR_{POF}+LR_{IND}\approx \chi_{2}^{2}. \end{array}$$ (51)

The hypothesis is $\mathrm{Pr}\left[I_{t}\left(1-q\right)=1|\Omega_{t-1}\right]=1-q,\forall_{t}$ against $\mathrm{Pr}\left[I_{t}\left(1-q\right)=1|\Omega_{t-1}\right]\neq 1-q,\forall_{t}$, where Ω_*t*−1_ is the information available on day *t* − 1. The model is rejected for the conditional coverage property if $LR_{CC}>\chi_{2}^{2}=5.99$.

The *DQ* test utilises the criterion that the number of exceptions produced on day *t* should be independent of the information available at day *t* − 1. The function is defined as

$$\begin{array}{c} {Hit}_{t}=I\left(L_{t}<-{VaR}_{q,t}^{p}\right)-\left(1-q\right)=\left\{ \begin{array}{c} q,\;\text{if}\;L_{t}<{VaR}_{q,t}^{p}\\ -(1-q),\;\text{otherwise}. \end{array}\right. \end{array}$$ (52)

The *Hit*_*t*_ function assumes the value *q* when the loss on the portfolio at time *t* is less than ${VaR}_{q,t}^{p}$, and −(1 − *q*) otherwise. As explained in [67], clearly E[*Hit*_*t*_] = 0, E[*Hit*_*t*_|Ω_*t*−1_] = 0 and *Hit*_*t*_ must be uncorrelated with its own lagged values. The test statistics is given by

$$\begin{array}{c} DQ=\frac{\left({Hit}_{t}^{{}^{\prime }}X_{t}{\left[X_{t}^{{}^{\prime }}X_{t}\right]}^{-1}X_{t}^{{}^{\prime }}{Hit}_{t}\right)}{(1-q)q}, \end{array}$$ (53)

where *X*_*t*_ is a vector containing all values of *Hit*_*t*_, ${VaR}_{q,t}^{p}$ and its lags. Under the null hypothesis E[*Hit*_*t*_] = 0 and E[*Hit*_*t*_|Ω_*t*−1_] = 0, *Hit*_*t*_ and *X*_*t*_ are orthogonal and *Hit*_*t*_ must be uncorrelated with its own lagged values [67, 71]. The *DQ* test is easy to perform, and does not depend on the estimation procedure; all that is needed is a series of VaRs and the corresponding values of the portfolio returns [67]. In this study, we follow [67, 72, 73] to use a constant, four lagged values of *Hit*_*t*_.

In Santos and Alves’ new class of independence test [68], we first define the duration between two consecutive exceptions as *D*_*i*_ = *t*_*i*_ − *t*_*i*−1_, where *t*_*i*_ denotes the time of exception number *i*; and *t*_0_ = 0 implies that *D*_1_ is the time until the first exception. We denote a sequence of *N* durations by ${\left\{D_{i}\right\}}_{i=1}^{N}$, where the order statistics are *D*_1:*N*_ ≤ … ≤ *D*_*N*:*N*_. The test statistics is defined as

$$\begin{array}{c} T_{N,[N/2]}=\log2\frac{D_{N:N}-1}{D_{[N/2]:N}}-\log N. \end{array}$$ (54)

See [68] for more details on this test.

Finally, BCBS developed a set of requirements that the VaR model must satisfy for it to be considered a reliable risk measure and proposed the Basel *traffic light* test. That is, (i) VaR must be calculated with 99% confidence, (ii) back-testing must be done using a minimum of a one year observation period and must be tested over at least 250 days, (iii) regulators should be 95% confident that they are not erroneously rejecting a valid VaR model, and (iv) Basel specifies a one-tailed test —it is only interested in the underestimation of risk [74]. [2] summarises the acceptance region for the Basel *traffic light* approach to back-testing VaR models.

We use out-of-sample data of *m* = *T* − *n* observations for back-testing; thus we have *n* = 1869 sample of the return observations for VaR estimation procedure containing the 2008 global financial crisis period, and *m* = 1000 of return observations for back-testing. VaR is then estimated following a rolling window approach. The out-of-sample data is further divided into blocks of 250, 500, and 1000 trading days to observe how the models behave for both longer and shorter observation periods. The division of out-of-sample data is also employed to meet the BCBS requirements. Table 10 presents the expected and observed number of exceptions produced following each model for a portfolio consisting of all five banks. At 99% confidence level and 250 trading days, the MS-GJR-GARCH(1,1) copula EVT VaR model registered 3 exceptions for a single-state and 0 exceptions for a two-state MS-GJR-GARCH(1,1) model. Thus, following Basel rules for back-testing, the VaR models passed the reliability test and are placed in the green zone. Back-testing results based on *LR*_*UC*_, *LR*_*IND*_, *LR*_*CC*_, *DQ*, and *T*_*N*,[*N*/2]_ tests are presented in Tables 11 and 12. For the *DQ* test, we use a lagged value of 4. In Tables 13 and 14 we present, as a benchmark for our VaR models, back-testing results for VaR models constructed using GJR-GARCH(1,1) and standard GARCH(1,1) (sGARCH(1,1)) volatility models with skewed Student’s-*t* errors but without copula functions and EVT. It can be seen from the number of exceptions recorded that the MS-GJR-GARCH(1,1) copula-EVT VaR model and the benchmark VaR models do not underestimate risk but rather too conservative at 99% and 95% confidence levels and thus preferred by most financial institutions. Most financial institutions will prefer VaR models with zero or very few exceptions as they routinely produce plots of *P*&*L* that show no violation of their 99% confidence VaR models over long periods stating that this supports their risk models. “The amount of economic capital banks currently hold is in excess of their regulatory capital. As a result, banks may prefer to report higher VaR numbers to avoid the possibility regulatory intrusion” [2]. Kupiec’s UC test (*LR*_*UC*_) will reject VaR models that produce 0 exceptions. The GJR-GARCH(1,1) copula-EVT VaR model captures VaR quite well in periods of calm and in periods of crisis for short and long observation periods. It does not overestimate or underestimate the level of risk on the portfolio and is does considered reliable as a measure of risk. Performance evaluation for rejection or acceptance of the VaR models based on 5% significance level are presented in Table 15. Clearly, the MS-GJR-GARCH(1,1) and GJR-GARCH(1,1) copula-EVT VaR models incorporating the *hybrid* method for threshold selection perform better than the standard GARCH(1,1) and GJR-GARCH(1,1) VaR models.

**Table 10:** Expected versus observed number of exceptions following Bayesian MS-GJR-GARCH(1,1) and GJR-GARCH(1,1) copula-EVT VaR model.

		250		500		1000	
		1%	5%	1%	5%	1%	5%
	Expected exceptions	2.5	12.5	5	25	10	50
GJR-GARCH(1,1)	Observed exceptions for *t*-copula	3	11	4	26	8	57
	Coverage rate	0.012	0.044	0.008	0.052	0.008	0.057
	Observed exceptions for Frank copula	3	11	5	24	9	55
	Coverage rate	0.012	0.044	0.010	0.048	0.009	0.055
MS-GJR-GARCH(1,1)	Observed exceptions for *t*-copula	0	6	0	15	0	33
	Coverage rate	0.000	0.024	0.000	0.030	0.000	0.033
	Observed exceptions for Frank copula	0	6	0	14	0	32
	Coverage rate	0.000	0.024	0.000	0.028	0.000	0.032

Note: Out-of-sample data is divided into blocks of 250, 500, and 1000 observation periods, time horizon of 1 day. The coverage rate $\frac{T_{1}}{T_{w}}\approx 1-q$.

**Table 11:** Back-testing results following Bayesian GJR-GARCH(1,1) Student’s-*t* and Frank copula-EVT VaR models.

**Student’s-*t* copula**			Back-test type				
Prob	Window	Exceptions	*LR*_*UC*_	*LR*_*IND*_	*LR*_*CC*_	*DQ*	*T*_*N*,[*N*/2]_
1%:	250	3	0.095 (0.758)	0.170 (0.680)	0.265 (0.876)	0.213 (0.999)	-0.072 (0.966)
	500	4	0.217 (0.641)	0.140 (0.708)	0.357 (0.837)	0.415 (0.998)	-0.616 (0.994)
	1000	8	0.434 (0.510)	0.268 (0.605)	0.702 (0.704)	1.057 (0.983)	1.386 (0.385)
5%:	250	11	0.197 (0.657)	2.475 (0.116)	2.672 (0.263)	2.057 (0.914)	2.108 (0.348)
	500	26	0.042 (0.838)	1.997 (0.158)	2.039 (0.361)	2.962 (0.814)	-0.254 (0.742)
	1000	57	0.989 (0.320)	3.145 (0.076)	4.134 (0.127)	7.494 (0.278)	-1.039 (0.939)
**Frank copula**			Back-test type				
Prob	Window	Exceptions	*LR*_*UC*_	*LR*_*IND*_	*LR*_*CC*_	*DQ*	*T*_*N*,[*N*/2]_
1%:	250	3	0.095 (0.758)	0.168 (0.682)	0.263 (0.877)	0.213 (0.999)	-0.072 (0.967)
	500	5	0.000 (1.000)	0.219 (0.640)	0.219 (0.896)	0.415 (0.998)	-0.839 (0.999)
	1000	9	0.105 (0.746)	0.340 (0.560)	0.445 (0.801)	1.066 (0.983)	0.537 (0.676)
5%:	250	11	0.197 (0.657)	1.887 (0.170)	2.084 (0.353)	2.057 (0.914)	2.426 (0.244)
	500	24	0.043 (0.836)	1.343 (0.247)	1.386 (0.500)	2.305 (0.890)	-0.174 (0.723)
	1000	55	0.510 (0.475)	2.531 (0.112)	3.041 (0.219)	6.335 (0.387)	-1.004 (0.934)

Note: *p*-values in parenthesis. Four lags were used for the *DQ* test.

**Table 12:** Back-testing results following Bayesian MS-GJR-GARCH(1,1) Student’s-*t* and Frank copula-EVT VaR models.

**Student’s-*t* copula**			Back-test type				
Prob	Window	Exceptions	*LR*_*UC*_	*LR*_*IND*_	*LR*_*CC*_	*DQ*	*T*_*N*,[*N*/2]_
1%:	250	0	*NaN*	-	-	0.213 (0.999)	-
	500	0	*NaN*	-	-	0.415 (0.998)	-
	1000	0	*NaN*	-	-	1.057 (0.983)	-
5%:	250	6	4.369 (0.037)	0.641 (0.423)	5.010 (0.082)	1.527 (0.958)	3.069 (0.379)
	500	15	4.884 (0.027)	0.001 (0.975)	4.885 (0.087)	2.226 (0.898)	1.154 (0.474)
	1000	33	6.878 (0.009)	0.032 (0.858)	6.910 (0.032)	0.697 (0.995)	-0.793 (0.908)
**Frank copula**			Back-test type				
Prob	Window	Exceptions	*LR*_*UC*_	*LR*_*IND*_	*LR*_*CC*_	*DQ*	*T*_*N*,[*N*/2]_
1%:	250	0	*NaN*	-	-	0.213 (0.999)	-
	500	0	*NaN*	-	-	0.415 (0.998)	-
	1000	0	*NaN*	-	-	1.057 (0.983)	-
5%:	250	6	4.369 (0.037)	0.699 (0.403)	5.068 (0.079)	30.724 (0.000)	2.070 (0.307)
	500	14	6.018 (0.014)	0.032 (0.858)	6.050 (0.049)	13.819 (0.032)	1.223 (0.382)
	1000	32	7.777 (0.005)	0.007 (0.933)	7.784 (0.020)	7.321 (0.292)	-1.386 (0.979)

Note: *p*-values in parenthesis, *NaN* = Not a Number. Four lags were used for the *DQ* test.

**Table 13:** Expected versus observed number of exceptions following sGARCH(1,1) and GJR-GARCH(1,1) models with skewed student’s-*t* errors.

	250		500		1000	
	1%	5%	1%	5%	1%	5%
Expected exceptions	2.5	12.5	5	25	10	50
Observed exceptions sGARCH(1,1)	0	7	0	16	0	32
Coverage rate	0.000	0.028	0.000	0.032	0.000	0.032
Observed exceptions GJR-GARCH(1,1)	0	7	0	14	0	26
Coverage rate	0.000	0.028	0.000	0.028	0.000	0.026

Note: Out-of-sample data is divided into blocks of 250, 500, and 1000 observation periods, time horizon of 1 day. The coverage rate $\frac{T_{1}}{T_{w}}\approx 1-q$.

**Table 14:** Back-testing results following sGARCH(1,1) and GJR-GARCH(1,1) models with skewed student’s-*t* errors.

**sGARCH**			Back-test type				
Prob	Window	Exceptions	*LR*_*UC*_	*LR*_*IND*_	*LR*_*CC*_	*DQ*	*T*_*N*,[*N*/2]_
1%:	250	0	*NaN*	-	-	37.933 (0.000)	-
	500	0	*NaN*	-	-	18.530 (0.005)	-
	1000	0	*NaN*	-	-	11.132 (0.084)	-
5%:	250	7	3.009 (0.083)	0.962 (0.327)	3.971 (0.137)	3.888 (0.692)	1.916 (0.448)
	500	16	3.888 (0.049)	0.011 (0.916)	3.899 (0.142)	2.409 (0.879)	-0.069 (0.708)
	1000	32	7.777 (0.005)	0.007 (0.933)	7.784 (0.020)	5.486 (0.483)	0.416 (0.541)
**GJR-GARCH(1,1)**			Back-test type				
Prob	Window	Exceptions	*LR*_*UC*_	*LR*_*IND*_	*LR*_*CC*_	*DQ*	*T*_*N*,[*N*/2]_
1%:	250	0	*NaN*	-	-	23.077 (0.001)	-
	500	0	*NaN*	-	-	48.651 (0.000)	-
	1000	0	*NaN*	-	-	95.686 (0.000)	-
5%:	250	7	3.009 (0.083)	0.962 (0.327)	3.971 (0.137)	57.759 (0.000)	4.119 (0.228)
	500	14	6.018 (0.014)	0.032 (0.858)	6.050 (0.049)	85.830 (0.000)	-0.773 (0.908)
	1000	26	14.597 (0.000)	0.263 (0.608)	14.860 (0.001)	169.533 (0.000)	-0.975 (0.928)

Note: *p*-values in parenthesis, *NaN* = Not a Number. Four lags were used for the *DQ* test.

**Table 15:** Performance Evaluation based on 5% significance level.

*P* = 1%	Back-test type					
VaR model	Window	*LR*_*UC*_	*LR*_*IND*_	*LR*_*CC*_	*DQ*	*T*_*N*,[*N*/2]_
GJR-GARCH(1,1) Student’s-*t* copula-EVT	250	A (0.758)	A (0.680)	A (0.876)	A (0.999)	A (0.966)
	500	A (0.641)	A (0.708)	A (0.837)	A (0.998)	A (0.994)
	1000	A (0.510)	A (0.605)	A (0.704)	A (0.983)	A (0.385)
GJR-GARCH(1,1) Frank copula-EVT	250	A (0.758)	A (0.682)	A (0.877)	A (0.999)	A (0.967)
	500	A (1.000)	A (0.640)	A (0.896)	A (0.998)	A (0.999)
	1000	A (0.320)	A (0.076)	A (0.127)	A (0.278)	A (0.939)
MS-GJR-GARCH (1,1) Student’s-*t* copula-EVT	250	R (*NaN*)	R (-)	R (-)	A (0.999)	R (-)
	500	R (*NaN*)	R (-)	R (-)	A (0.998)	R (-)
	1000	R (*NaN*)	R (-)	R (-)	A (0.983)	R (-)
MS-GJR-GARCH(1,1) Frank copula-EVT	250	R (*NaN*)	R (-)	R (-)	A (0.999)	R (-)
	500	R (*NaN*)	R (-)	R (-)	A (0.998)	R (-)
	1000	R (*NaN*)	R (-)	R (-)	A (0.983)	R (-)
sGARCH(1,1)	250	R (*NaN*)	R (-)	R (-)	R (0.000)	R (-)
	500	R (*NaN*)	R (-)	R (-)	R (0.005)	R (-)
	1000	R (*NaN*)	R (-)	R (-)	A (0.084)	R (-)
GJR-GARCH(1,1)	250	R (*NaN*)	R (-)	R (-)	R (0.001)	R (-)
	500	R (*NaN*)	R (-)	R (-)	R (0.000)	R (-)
	1000	R (*NaN*)	R (-)	R (-)	R (0.000)	R (-)
*P* = 5%	Back-test type					
VaR model	Window	*LR*_*UC*_	*LR*_*IND*_	*LR*_*CC*_	*DQ*	*T*_*N*,[*N*/2]_
GJR-GARCH(1,1) Student’s-*t* copula-EVT	250	A(0.657)	A(0.116)	A(0.263)	A(0.914)	A(0.348)
	500	A (0.838)	A (0.158)	A (0.361)	A (0.814)	A (0.742)
	1000	A (0.320)	A (0.076)	A (0.127)	A (0.278)	A (0.939)
GJR-GARCH(1,1) Frank copula-EVT	250	A (0.657)	A (0.170)	A (0.353)	A (0.914)	A (0.244)
	500	A (0.836)	A (0.247)	A (0.500)	A (0.890)	A (0.723)
	1000	A (0.475)	A (0.112)	A (0.219)	A (0.387)	A (0.934)
MS-GJR-GARCH(1,1) Student’s-*t* copula-EVT	250	R (0.037)	A (0.423)	A (0.082)	A (0.958)	A (0.379)
	500	R (0.027)	A (0.975)	A (0.087)	A (0.898)	A (0.474)
	1000	R (0.009)	A (0.858)	R (0.032)	A (0.995)	A (0.908)
MS-GJR-GARCH(1,1) Frank copula-EVT	250	R (0.037)	A (0.403)	A (0.079)	R (0.000)	A (0.307)
	500	R (0.014)	A (0.858)	R (0.049)	R (0.032)	A (0.382)
	1000	R (0.005)	A (0.933)	R (0.020)	A (0.292)	A (0.979)
sGARCH(1,1)	250	A (0.083)	A (0.327)	A (0.137)	A (0.692)	A (0.448)
	500	R (0.049)	A (0.916)	A (0.142)	A (0.879)	A (0.708)
	1000	R (0.005)	A (0.933)	R (0.020)	A (0.483)	A (0.541)
GJR-GARCH(1,1)	250	A (0.083)	A (0.327)	A (0.137)	R (0.000)	A (0.228)
	500	R (0.014)	A (0.858)	R (0.049)	R (0.000)	A (0.908)
	1000	R (0.000)	A (0.608)	R (0.001)	R (0.000)	A (0.928)

Note: A = Accept, R = Reject, *p*-values in parenthesis. *NaN* = Not a Number; as a result of zero exceptions.

## Conclusion

In recent decades, VaR has become the most common risk measure used by financial institutions to assess market risk of financial assets. Since VaR models often focus on the behavior of asset returns in the left tail, it is important that the models are calibrated such that they do not underestimate or overestimate the proportion of outliers, as this will have significant effects on the allocation of economic capital for investments. Due to the *extremistan* [75] nature of financial asset returns and volatility, the real tail risk of a financial asset is not stable as time passes, and the maximum loss is difficult to predict. Therefore, to implement a reliable VaR model, the time horizon and type of volatility model used is very important. We constructed our VaR models by combining a single-state and two-state Bayesian MS-GJR-GARCH(1,1) models with skewed Student’s-*t* distributions for the underlying volatility model, copula functions to model dependence, and EVT to model the left tail. The single-state Bayesian MS-GJR-GARCH(1,1) is a GJR-GARCH(1,1) model without regime change, hence the names Bayesian GJR-GARCH(1,1) copula-EVT VaR model for the single-state MS-GJR-GARCH(1,1) and Bayesian MS-GJR-GARCH(1,1) copula-EVT VaR model for the two-state MS-GJR-GARCH(1,1).

We use as a benchmark, VaR models constructed using GJR-GARCH(1,1) and sGARCH(1,1) volatility models with skewed Student’s-*t* distributions, but without copula functions and EVT to compare the performance of our VaR models. Back-testing results show that the GJR-GARCH(1,1) copula-EVT VaR model is much reliable than the MS-GJR-GARCH(1,1) copula EVT VaR model and the benchmark VaR models. Back-testing results further indicates that the GJR-GARCH(1,1) copula EVT VaR model does not overestimate or underestimate the level of risk on the portfolio whereas the two-state MS-GJR-GARCH(1,1) copula EVT VaR model and the benchmark VaR models seems to overestimate the level of risk.

It is also important to draw attention to the fact that Eq (34) is a point estimate with an error band that becomes larger as we move to more extreme quantiles. It is concerned only with the number of exceedances above a certain threshold and is not affected by data outside the tail of the distribution [8]. This can be problematic in some cases due to limited data points in the tail, which can inhibit proper analysis.

Eq (34) also depends on the threshold and the number of points (i.e., exceedances) above the threshold because the parameters are estimated based on the exceedances. Thus, it is logical to say that the reliability of Eq (34) rests solely on the choice of thresholds, which is subjective. The proposed *hybrid* method for the threshold addresses this issue and diminishes the possibility of selecting a less suitable threshold value. This method is reliable and can be implemented with other conditional multivariate volatility models providing positive-definite volatility matrices.
